# Clinical burden of illness in patients with phenylketonuria (PKU) and associated comorbidities - a retrospective study of German health insurance claims data

**DOI:** 10.1186/s13023-019-1153-y

**Published:** 2019-07-22

**Authors:** K. F. Trefz, A. C. Muntau, K. M. Kohlscheen, J. Altevers, C. Jacob, S. Braun, W. Greiner, A. Jha, M. Jain, I. Alvarez, P. Lane, C. Schröder, F. Rutsch

**Affiliations:** 1 Zentrum für Kinder- und Jugendmedizin, Universitätsklinikum Heidelberg, Heidelberg, Germany; 2 University Children’s Hospital, University Medical Center Hamburg-Eppendorf, Hamburg, Germany; 3 Xcenda GmbH, Hannover, Germany; 4 Fakultät für Gesundheitswissenschaften, Universität Bielefeld, Bielefeld, Germany; 5 BioMarin Europe Ltd., London, UK; 6 BioMarin Deutschland GmbH, Kronberg/Ts, Germany; 7 Kinder- und Jugendmedizin – Allgemeine Pädiatrie, Universitätsklinikum Münster, Münster, Germany

**Keywords:** Phenylketonuria, Burden of illness, Burden of disease, Claims data, Statutory health insurance, Hyperphenylalaninemia

## Abstract

**Background:**

Phenylketonuria (PKU) is an inherited deficiency in the enzyme phenylalanine hydroxylase (PAH), which, when poorly-managed, is associated with clinical features including deficient growth, microcephaly, seizures, and intellectual impairment. The management of PKU should start as soon as possible after diagnosis to prevent irreversible damage and be maintained throughout life. The aim of this study was to assess the burden of illness in PKU patients in general and in PKU patients born before and after the introduction of newborn screening in Germany.

**Methods:**

This retrospective matched cohort analysis used the Institut für angewandte Gesundheitsforschung Berlin (InGef) research database containing anonymized healthcare claims of approximately 4 million covered lives. PKU patients were compared with matched controls from the general population within the same database (1:10 ratio via direct, exact matching on age and gender without replacement). PKU patients were included if they were aged ≥18 years on 01/01/15 and were continuously enrolled from 01/01/10 to 31/12/15. The 50 most commonly reported comorbidities and 50 most commonly prescribed medications in the PKU population were analyzed. Differences between groups were tested using 95% confidence interval (CI) of prevalence ratio (PR) values.

**Results:**

The analysis included 377 adult PKU patients (< 5 of which were receiving sapropterin dihydrochloride) and 3,770 matched controls. Of the 50 most common comorbidities in the PKU population, those with a statistically significant PR > 1.5 vs controls included major depressive disorders (PR = 2.3), chronic ischemic heart disease (PR = 1.7), asthma (PR = 1.7), dizziness and giddiness (PR = 1.8), unspecified diabetes mellitus (PR = 1.7), infectious gastroenteritis and colitis (PR = 1.7), and reaction to severe stress and adjustment disorders (PR = 1.6). The most commonly prescribed Anatomical Therapeutic Chemical (ATC) subcodes among PKU patients (vs the control population) are for systemic antibacterials (34.7% vs 32.8%), anti-inflammatory and antirheumatic (29.4% vs 27.5%), renin-angiotensin agents (30.0% vs 27.0%), acid-related disorders (29.4% vs 20.2%), and beta-blockers (24.9% vs 19.9%).

**Conclusion:**

The overall clinical burden on patients with PKU is exacerbated by a significantly higher risk of numerous comorbidities and hence, prescribing of the requisite medication, both for recognized (e.g. major depressive disorders) and more unexpected comorbidities (e.g. ischemic heart disease).

**Electronic supplementary material:**

The online version of this article (10.1186/s13023-019-1153-y) contains supplementary material, which is available to authorized users.

## Background

Phenylketonuria (PKU) is, in 98–99% of cases, due to an inherited deficiency in the enzyme phenylalanine hydroxylase (PAH), which results in elevated levels of the essential amino acid phenylalanine (Phe) and reduced levels of tyrosine [1]. PKU is caused by over 1,000 different gene variants of PAH [2] and the severity of the resulting disease ranges from mild to severe, based on the residual enzyme activity and the level of Phe circulating in the blood (blood Phe) [1, 3]. High blood Phe levels alter large neutral amino acid (e.g. tyrosine, tryptophan) transfer across the blood-brain barrier and interfere with the production of neurotransmitters. To this end, high blood and brain Phe concentrations in patients with PKU are associated with deleterious effects on neurocognitive outcomes [3].

Management of PKU should be maintained throughout life and should start as soon as possible after diagnosis via newborn screening (NBS) to prevent irreversible damage, such as neurological impairment and mental retardation [4, 5]. Besides the start of an early treatment, strict blood Phe control is of primary importance for an optimal outcome, particularly during the first years of life [5]. The management of PKU comprises the reduction of dietary intake of Phe by low-protein diets and Phe-free amino acid supplements, and may include low-protein supplements/foods. Additionally, sapropterin dihydrochloride (sapropterin, Kuvan^®^, BioMarin Pharmaceutical Inc., Novato, CA, USA), a synthetic version of BH4, the naturally occurring co-factor of PAH, can be used in responsive patients to stimulate residual PAH activity [6, 7]. Dietary management options are ineffective in many adults with PKU due to long-term adherence issues [8–10] or inadequate Phe-lowering effects [6]. Moreover, a longtime Phe-restricted diet is associated with vitamin and/or mineral deficiencies [11, 12].

The impact of the disease on individual patients and the healthcare system as a whole can only be understood when considering all associated comorbidities that affect patients. PKU is often associated with neuropsychiatric, behavioral and cognitive symptoms, but the full range of systemic comorbidities associated with PKU and long-term exposure to elevated blood Phe are poorly understood.

The aim of this study was to assess the comorbidity profile of adult PKU patients in Germany and gain insights into the burden of illness in PKU patients.

## Results

### Patient populations and general health

Overall, 3,723,345 individuals in the InGef research database were continuously enrolled during the study period from January 1st, 2015 until December 31st, 2015. Thereof, 377 adult individuals with PKU were identified, resulting in a period prevalence of 10.13 in 2015 (per 100,000 individuals). Most adult PKU patients were female (58.1%) and the mean age of adult PKU patients in 2015 was 50.9 ± 20.4 years (Table 1).

**Table 1:** Age and gender of PKU patients in total PKU population, early-diagnosed, and late-diagnosed patients

		PKU patients (*n* = 377)	Early-diagnosed PKU patients (*n* = 161)	Late-diagnosed PKU patients (*n* = 216)
Gender	Female, n (%)	219 (58.1)	101 (62.7)	118 (54.6)
	Male, n (%)	158 (41.9)	60 (37.2)	98 (45.4)
Age (years)	Mean (SD)	50.9 (20.4)	30.7 (8.2)	65.9 (12.1)
	Median	51	30	65
	Range	18–96	18–46	46–96

From the 377 patients in the adult PKU cohort, 161 (42.7%) patients were born in 1969 (implementation of NBS) or later (presumed to be early-diagnosed) and 216 (57.3%) patients were born prior to 1969 (presumed to be late-diagnosed). Due to this classification by birth year, the mean age of early-diagnosed patients (30.7 ± 8.2 years) was less than half that of the late-diagnosed patients (65.9 ± 12.1 years; Table 1). Additionally, there was a higher proportion of females in the early-diagnosed group (*n* = 101; 62.7%) than in the late-diagnosed group (*n* = 118; 54.6%). Less than 1.3% of the overall population were receiving sapropterin (< 5 patients; specific number not identified in this study due to patient privacy). All patients receiving sapropterin were early-diagnosed patients. While 52 (13.8%) patients in the overall PKU population were receiving D.A.S. (Phe-free Dietary Amino Acid Supplement), these were mainly in the early-diagnosed group (*n* = 47, 29.2% of early-diagnosed patients vs *n* = 5, 2.3% of the late-diagnosed patients).

When assessing the Updated Charlson Comorbidity Index (CCI) for the adult PKU cohort, the PKU cohort shows a higher burden of the CCI constituent comorbidities compared to the matched cohort (Table 2). The PKU cohort shows significantly more comorbid burden than controls (20.2% vs 13.1% with CCI scores ≥3). The late-diagnosed PKU patients have a significantly higher comorbid burden compared with their matched controls, especially in terms of severity (33.8% vs 22.3% of subjects had a CCI score ≥ 3; CCI categories among the late-diagnosed PKU cohort and the matched cohort are shown in Additional file 1: Table S1).

**Table 2:** Updated CCI categories among the PKU cohort and the matched cohort

	PKU patients (*n* = 377)		Control group (*n* = 3,770)		Chi^2^ test *p value*	PR (95% CI)
	*n*	%	*n*	%		
CCI = 0	194	51.5	2,206	58.5	0.001	0.9 (0.79–0.97)
CCI = 1	72	19.1	716	19.0	1.000	1.0 (0.81–1.25)
CCI = 2	35	9.3	353	9.4	1.000	1.0 (0.71–1.38)
CCI = 3	30	8.0	189	5.0	0.021	1.6 (1.10–2.30)
CCI ≥ 4	46	12.2	306	8.1	0.009	1.5 (1.12–2.01)

CCI of 0 = no comorbidities, ≥4 = severe comorbidities

There were no significant differences in comorbid burden between early-diagnosed PKU patients and their matched controls (Additional file 1: Table S2). Unsurprisingly, given the markedly younger age of the early-diagnosed cohort (mean age 30.7 years), they had a lower comorbid burden than the late-diagnosed cohort (mean age 65.9 years) and no patients had a CCI score ≥ 3 (vs 33.8% in the late-diagnosed cohort).

### Comorbidity profile

#### Adult PKU patients

The analysis included 377 adult patients with PKU and 3,770 matched control subjects. The most common comorbidities were assessed by identifying the 50 most prevalent comorbidities among adult PKU patients in 2015 in the database. More than a third (38.7%) of adult PKU patients suffered from essential (primary) hypertension, dorsalgia (35.3%), and disorders of lipoprotein metabolism and other lipidemias (33.7%). The full list of the 50 most prevalent comorbidities is shown in Additional file 1: Table S3 and those that were present in > 10% of the adult PKU patients are shown in Table 3.

**Table 3:** Comorbidity profile^a^ of adult PKU patients and matched controls in Germany in 2015

ICD-10-GM code	Comorbidity	PKU population (*n* = 377)	Control population (*n* = 3,770)	PR (95% CI)
		%	%	
I10	Essential (primary) hypertension	38.7	36.2	1.07 (0.94, 1.22)
M54	Dorsalgia	35.3	30.3	1.16 (1.01, 1.34) ^b^
E78	Disorders of lipoprotein metabolism and other lipidemias	33.7	25.1	1.34 (1.15, 1.56) ^b^
Z12	Encounter for screening for malignant neoplasms	31.3	30.0	1.04 (0.89, 1.22)
H52	Disorders of refraction and accommodation	27.3	23.6	1.16 (0.97, 1.38)
Z30	Encounter for contraceptive management	22.5	21.2	1.06 (0.87, 1.30)
N89	Other noninflammatory disorders of vagina	21.8	18.0	1.21 (0.99, 1.48)
J06	Acute upper respiratory infections of multiple and unspecified sites	21.5	18.8	1.14 (0.93, 1.40)
Z01	Encounter for other specified exam without complaint, suspected or reported dx	20.7	14.0	1.47 (1.19, 1.82) ^b^
F32	Major depressive disorder, single episode	16.2	13.3	1.22 (0.95, 1.55)
Z00	Encounter for general exam without complaint, suspected or reported dx	15.9	14.4	1.11 (0.87, 1.42)
E66	Overweight and obesity	15.9	11.2	1.43 (1.11, 1.83) ^b^
I25	Chronic ischemic heart disease	15.7	9.0	1.74 (1.35, 2.25) ^b^
F45	Somatoform disorders	15.4	12.1	1.27 (0.98, 1.63)
E11	Type 2 diabetes mellitus	14.9	11.6	1.28 (0.99, 1.66)
Z25	Need for immunization against other single viral diseases	14.3	13.1	1.09 (0.84, 1.42)
M53	Other and unspecified dorsopathies, not elsewhere classified	14.3	10.2	1.40 (1.08, 1.83) ^b^
E04	Other nontoxic goiter	14.1	10.8	1.30 (1.00, 1.69)
M47	Spondylosis	13.8	12.2	1.13 (0.86, 1.47)
R10	Abdominal and pelvic pain	13.3	11.4	1.17 (0.89, 1.53)
M17	Osteoarthritis of knee	13.3	10.1	1.31 (1.00, 1.73)
N39	Other disorders of urinary system	13.0	9.4	1.38 (1.04, 1.82) ^b^
K29	Gastritis and duodenitis	12.7	9.7	1.32 (0.99, 1.75)
N95	Menopausal and other perimenopausal disorders	12.5	8.6	1.46 (1.09, 1.94) ^b^
J45	Asthma	11.9	7.0	1.70 (1.26, 2.29) ^b^
D22	Melanocytic nevi	11.7	9.2	1.28 (0.95, 1.71)
E03	Other hypothyroidism	11.7	9.5	1.23 (0.92, 1.66)
J30	Vasomotor and allergic rhinitis	11.7	8.8	1.32 (0.98, 1.78)
R42	Dizziness and giddiness	11.1	6.0	1.84 (1.35, 2.52) ^b^
F43	Reaction to severe stress, and adjustment disorders	10.9	7.0	1.56 (1.15, 2.14) ^b^
E14	Unspecified diabetes mellitus	10.9	6.4	1.69 (1.23, 2.31) ^b^
A09	Infectious gastroenteritis and colitis, unspecified	10.6	6.3	1.69 (1.23, 2.33) ^b^
M79	Other and unspecified soft tissue disorders, not elsewhere classified	10.6	7.2	1.48 (1.08, 2.03) ^b^
L30	Other and unspecified dermatitis	10.6	7.3	1.44 (1.05, 1.98) ^b^
L30	Personal history of medical treatment	10.6	7.7	1.38 (1.01, 1.89) ^b^
Q66	Congenital deformities of feet	10.3	8.0	1.29 (0.94, 1.77)
I83	Varicose veins of lower extremities	10.3	8.8	1.18 (0.86, 1.62)
E79	Disorders of purine and pyrimidine metabolism	10.1	6.8	1.49 (1.08, 2.06) ^b^
T78	Adverse events, not elsewhere classified	10.1	5.9	1.71 (1.23, 2.37) ^b^

^a^Only comorbidities present in > 10% of PKU patients are shown; a full listing of the top 50 comorbidities is provided in Additional file 1: Table S3

^b^Comorbidities that had a significant PR vs the control population

**Fig. 1:**
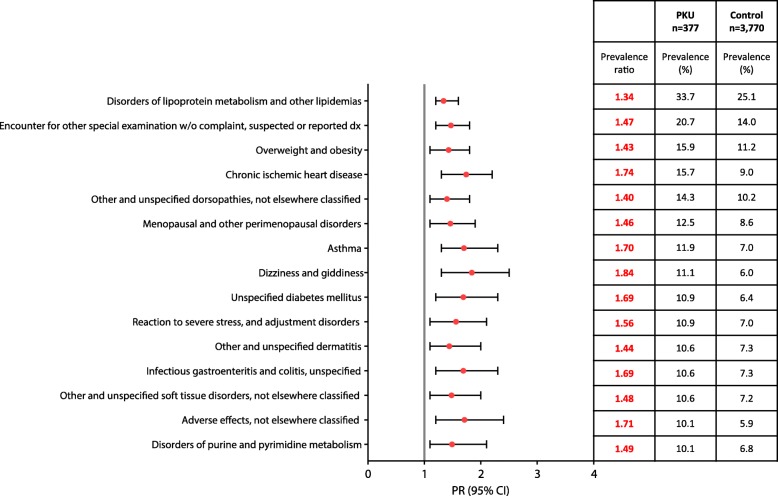
Comorbidities with a prevalence > 10% among the 50 most frequent that are significantly more prevalent in the PKU vs control population

Among the comorbidities that were present in > 10% of the PKU cohort, those that were significantly more prevalent in the PKU vs control population included: chronic ischemic heart disease (Prevalence = 15.7%; PR = 1.7; 95% CI 1.35–2.25); asthma (Prevalence = 11.9%; PR = 1.7; 95% CI 1.26–2.29); dizziness and giddiness (Prevalence = 11.1%; PR = 1.8; 95% CI 1.35–2.52); unspecified diabetes mellitus (Prevalence = 10.9%; PR = 1.7; 95% CI 1.23–2.31); reaction to severe stress and adjustment disorders (Prevalence = 10.9%; PR = 1.6; 95% CI 1.15–2.14); infectious gastroenteritis and colitis (Prevalence = 10.6%; PR = 1.7; 95% CI 1.23–2.33); and adverse effects, not elsewhere classified (Prevalence = 10.1%; PR = 1.7; 95% CI 1.23–2.37) (Fig. 1). Additionally, comorbidities in the top 50 most common in the PKU population with a PR > 1.5 for the PKU cohort compared with the matched controls, but not shown in Table 3 (i.e. present in < 10% of PKU patients), included other acquired deformities of limbs (Prevalence = 9.8%; PR = 2.2; 95% CI 1.60–3.15), other chronic obstructive pulmonary disease (Prevalence = 9.5%; PR = 1.9; 95% CI 1.34–2.65), other anxiety disorders (Prevalence = 9.3%; PR = 1.8; 95% CI 1.29–2.56), and major depressive disorders, recurrent (Prevalence = 8.8%; PR = 2.3; 95% CI 1.57–3.25).

The 50 most frequently prescribed agents in PKU patients are provided in full in Additional file 1: Table S4 and those prescribed in > 10% of PKU patients in Table 4. The most common Anatomical Therapeutic Chemical (ATC) categories of prescribed agents that are significantly more prevalent in PKU vs controls are cardiovascular (43.8% vs 37.4%; PR 1.17; 1.04, 1.32), nervous system (40.3% vs 28.4%; PR 1.42; 95% CI 1.24, 1.62), alimentary tract and metabolism (40.6% vs 29.6%; PR 1.37; 95% CI 1.20, 1.56), and dermatologicals (22.0% vs 15.5%; PR 1.41; 95% CI 1.15, 1.73). The most common ATC subcodes for the prescribed agents with significant differences between the PKU and control populations are for acid-related disorders (29.4% vs 20.2%; PR 1.46; 95% CI 1.23, 1.72) and analgesics (24.4% vs 19.0%; PR 1.28; 95% CI 1.06, 1.55). Additionally, beta-blockers, lipid-modifying agents, diuretics, calcium channel blockers, cardiac therapy, vitamins, minerals, pyschoanaleptics, psycholeptics, antiepileptics, other nervous system drugs, vaccines, antigout preparations, corticosteroids for systemic use, corticosteroid dermatological preparations, antimycotics for dermatological use, and gynecological antiinfectives and antiseptics were all prescribed significantly more often in the PKU vs control populations.

**Table 4:** ATC codes of the top 50 most commonly prescribed agents in the PKU population^a^

ATC code or subcode	Category or subcategory	PKU patients (*n* = 377) %	Control group (*n* = 3,770) %	PR (95% CI)
**C**	**Cardiovascular system**	**43.8**	**37.4**	**1.17 (1.04, 1.32)** ^b^
C09	Agents acting on the renin-angiotensin system	30.0	27.0	1.11 (0.94, 1.31)
C07	Beta blocking agents	24.9	19.9	1.25 (1.04, 1.51) ^b^
C10	Lipid modifying agents	19.4	13.1	1.48 (1.19, 1.85) ^b^
C03	Diuretics	15.9	10.4	1.53 (1.19, 1.97) ^b^
C08	Calcium channel blockers	12.5	8.9	1.40 (1.05, 1.86) ^b^
C01	Cardiac therapy	6.1	3.1	1.95 (1.26, 3.01) ^b^
**A**	**Alimentary tract and metabolism**	**40.6**	**29.6**	**1.37 (1.20, 1.56)** ^b^
A02	Drugs for acid related disorders	29.4	20.2	1.46 (1.23, 1.72) ^b^
A11	Vitamins	4.5	2.0	2.27 (1.35, 3.80) ^b^
A12	Minerals	3.7	1.3	2.86 (1.59, 5.13) ^b^
**N**	**Nervous system**	**40.3**	**28.4**	**1.42 (1.24, 1.62)** ^b^
N02	Analgesics	24.4	19.0	1.28 (1.06, 1.55) ^b^
N06	Psychoanaleptics	17.2	9.5	1.82 (1.43, 2.31) ^b^
N05	Psycholeptics	8.8	5.4	1.62 (1.14, 2.30) ^b^
N03	Antiepileptics	5.3	2.9	1.82 (1.14, 2.89) ^b^
N07	Other nervous system drugs	2.7	1.3	2.08 (1.06, 4.08) ^b^
**J**	**Antiinfectives for systemic use**	**36.6**	**34.0**	**1.08 (0.94, 1.24)**
J01	Antibacterials for systemic use	34.7	32.8	1.06 (0.92, 1.23)
J07	Vaccines	1.9	0.3	6.36 (2.48, 16.32) ^b^
**M**	**Musculo-skeletal system**	**35.3**	**32.0**	**1.10 (0.95, 1.27)**
M01	Antiinflammatory and antirheumatic products	29.4	27.5	1.07 (0.91, 1.26)
M04	Antigout preparations	7.7	4.9	1.58 (1.09, 2.31) ^b^
**H**	**Systemic hormonal preparations, excl. Sexual hormones and insulins**	**24.7**	**20.6**	**1.20 (0.99, 1.44)**
H03	Thyroid therapy	16.7	15.3	1.09 (0.86, 1.39)
H02	Corticosteroids for systemic use	10.3	6.9	1.51 (1.09, 2.07) ^b^
**D**	**Dermatologicals**	**22.0**	**15.6**	**1.41 (1.15, 1.73)** ^b^
D07	Corticosteroid, dermatological preparations	12.5	9.0	1.39 (1.04, 1.85) ^b^
D01	Antimycotics for dermatological use	5.6	3.5	1.58 (1.01, 2.47) ^b^
**R**	**Respiratory system**	**21.5**	**16.6**	**1.30 (1.06, 1.59)** ^b^
R03	Drugs for obstructive airway diseases	13.0	9.8	1.32 (1.00, 1.74)
**G**	**Genitourinary system and sexual hormones**	**17.2**	**11.6**	**1.49 (1.17, 1.89)** ^b^
G01	Gynecological antiinfectives and antiseptics	3.2	1.4	2.31 (1.24, 4.28) ^b^
**B**	**Blood and blood forming organs**	**16.2**	**14.5**	**1.12 (0.88, 1.42)**
B01	Antithrombotic agents	13.3	12.0	1.10 (0.84, 1.45)
**S**	**Sensory organs**	**8.8**	**11.4**	**0.77 (0.55, 1.08)**

^a^Data are only shown for subcodes that were prescribed in > 10% of PKU patients or those that were significant vs the control population. ATC category totals may not add up because subcategories with < 5 patients in the PKU group have been excluded from the table, but may still count to the total for the ATC class of drug

^b^ATC codes that had a significant PR vs the control population

One letter ATC codes (e.g. Dermatologicals, Cardiovascular system) are shown in bold

#### Early-diagnosed adult PKU patients

Twenty-one of the top 50 comorbidities in the early-diagnosed PKU patients were present in > 10% of the population and are shown in Table 5. The most common recorded ICD-10-GM codes among the early-diagnosed PKU patients were encounters for contraceptive management (Prevalence = 46.6%) and screening for malignant neoplasms (Prevalence = 35.4%). Furthermore, other noninflammatory disorders of vagina is among the top 3 most frequently recorded ICD-10-GM codes. There was a higher proportion of female patients among early-diagnosed adult PKU patients and there were more female-specific conditions in this population, such as for contraception. However, none of these conditions were significantly different between early-diagnosed PKU patients and their matched control group.

**Table 5:** Comorbidity profile^a^ of early-diagnosed adult PKU patients in 2015 in Germany

ICD-10-GM code	Comorbidity	Early-diagnosed PKU population (*n* = 161) %	Control population (*n* = 1,610) %	PR (95% CI)
Z30	Encounter for contraceptive management	46.6	42.4	1.10 (0.92, 1.31)
Z12	Encounter for screening for malignant neoplasms	35.4	30.1	1.18 (0.94, 1.47)
N89	Other noninflammatory disorders of vagina	31.7	27.8	1.14 (0.90, 1.45)
J06	Acute upper respiratory infections of multiple and unspecified sites	29.2	27.6	1.06 (0.82, 1.36)
M54	Dorsalgia	26.1	22.6	1.15 (0.88, 1.52)
Z01	Encounter for other specified exam without complaint, suspected or reported dx	23.6	15.5	1.52 (1.13, 2.05) ^b^
N94	Pain and other condition associated with female genital organs and menstrual cycle	18.0	12.9	1.39 (0.98, 1.98)
R10	Abdominal and pelvic pain	15.5	13.0	1.19 (0.81, 1.74)
J30	Vasomotor and allergic rhinitis	14.9	11.4	1.30 (0.88, 1.93)
A09	Infectious gastroenteritis and colitis, unspecified	14.9	9.9	1.51 (1.01, 2.25) ^b^
N92	Excessive, frequent and irregular menstruation	14.3	10.7	1.34 (0.89, 2.00
F43	Reaction to severe stress, and adjustment disorders	13.7	7.8	1.75 (1.14, 2.67) ^b^
H52	Disorders of refraction and accommodation	13.0	10.8	1.21 (0.79, 1.84)
F45	Somatoform disorders	13.0	9.3	1.40 (0.91, 2.15)
F32	Major depressive disorder, single episode	12.4	9.9	1.25 (0.81, 1.93)
E66	Overweight and obesity	11.8	7.1	1.67 (1.05, 2.63) ^b^
M79	Other and unspecified soft tissue disorders, not elsewhere classified	11.2	5.5	2.05 (1.27, 3.31) ^b^
F41	Other anxiety disorders	10.6	5.3	2.00 (1.22, 3.28) ^b^
M99	Biomechanical lesions, not elsewhere classified	10.6	8.7	1.21 (0.75, 1.96)
T78	Adverse effects, not elsewhere classified	10.6	6.8	1.56 (0.96, 2.53)
D22	Melanocytic nevi	10.6	8.9	1.19 (0.74, 1.91)

^a^ Only comorbidities present in > 10% of PKU Patients are shown; a full listing of the top 50 comorbidities is provided in Additional file 1: Table S5

^b^Comorbidities that had a significant PR vs the control population

Among the 21 most frequently coded ICD-10-GM-codes that occurred in > 10% of the early-diagnosed PKU population, those with a significant PR were: encounter for other specified examinations without complaint, suspected or reported diagnosis (Prevalence = 23.6%; PR = 1.52; 95% CI 1.13–2.05); infectious gastroenteritis and colitis (Prevalence = 14.9%; PR = 1.51; 95% CI 1.01–2.25); reaction to severe stress and adjustment disorders (Prevalence = 13.7%, PR = 1.7; 95% CI 1.14–2.67); overweight and obesity (Prevalence = 11.8%; PR = 1.7; 95% CI 1.05–2.63); other and unspecified soft tissue disorders, not elsewhere classified (Prevalence = 11.2%, PR = 2.0; 95% CI 1.27–3.31); and unspecified and other anxiety disorders (Prevalence = 10.6%; PR = 2.0; 95% CI 1.22–3.28). A complete list of the 50 most prevalent comorbidities among early-diagnosed PKU patients is provided in Additional file 1: Table S5.

Among the remainder of the top 50 comorbidities (Additional file 1: Table S5), those with a significant PR > 1.5 for the early-diagnosed PKU population vs matched controls were: hypotension (Prevalence = 6.2%; PR = 2.78; 95% CI 1.40–5.49); encounter for other consultation and medical advice (Prevalence = 6.8%; PR = 2.3; 95% CI 1.24–4.42); thoracic, thoracolumbar, and lumbosacral intervertebral disc disorders (Prevalence = 7.5%; PR = 2.2; 95% CI 1.19–3.99); major depressive disorder, recurrent (Prevalence = 6.8%: PR = 2.1; 95% CI 1.11–3.89); dizziness and giddiness (Prevalence = 6.2%; PR = 2.0; 95% CI 1.05–3.95); scoliosis (Prevalence = 6.8; PR = 2.0; 95% CI 1.09–3.82); disorders of lipoprotein metabolism and other lipidemias (Prevalence = 8.7%; PR = 1.8; 95% CI 1.07–3.18); need for immunization against combinations of infectious diseases (Prevalence = 8.1%; PR = 1.8; 95% CI 1.01–3.14); and acute tonsillitis (Prevalence = 9.9%; PR = 1.7; 95% CI 1.03–2.82).

The most common ATC categories of prescribed agents that are significantly more prevalent in early-diagnosed PKU population vs controls are (Table 6): nervous system (26.7% vs 17.8%; PR 1.50; 95% CI 1.14, 1.98), alimentary tract and metabolism (24.8% vs 14.0%; PR 1.78; 95% CI 1.32, 2.39), and cardiovascular (12.4% vs 6.3%; PR 1.98; 95% CI 1.26, 3.11). The ATC subcodes for the prescribed agents with significant differences between the early-diagnosed and control populations are for acid-related disorders (16.1% vs 9.3%; PR 1.73; 95% CI 1.18, 2.54), systemic corticosteroids (6.8% vs 3.4%; PR 2.00; 95% CI 1.07, 3.74), vitamins (5.6% vs 0.6%; PR 9.0; 95% CI 3.71, 21.8), and diuretics (3.1% vs 0.3%; PR 10.0; 95% CI 2.93, 34.18). A full listing of the top 50 ATC codes is provided in Additional file 1: Table S6.

**Table 6:** Top 50 most commonly prescribed ATC codes in the early-diagnosed PKU population^a^

ATC code or subcode	Category or subcategory	Early-diagnosed PKU patients (*n* = 161) %	Control group (*n* = 1,610) %	PR (95% CI)
**J**	**Antiinfectives for systemic use**	**34.8**	**34.7**	**1.00 (0.80, 1.25)**
J01	Antibacterials for systemic use	32.9	33.9	0.97 (0.77, 1.22)
**N**	**Nervous system**	**26.7**	**17.8**	**1.50 (1.14, 1.98)** ^b^
N06	Psychoanaleptics	14.3	6.1	2.35 (1.54, 3.59) ^b^
N02	Analgesics	12.4	11.3	1.10 (0.71, 1.69)
N05	Psycholeptics	8.7	2.2	3.89 (2.14, 7.06) ^b^
N03	Antiepileptics	3.7	0.7	5.00 (1.90, 13.14) ^b^
**M**	**Musculo-skeletal system**	**25.5**	**22.5**	**1.13 (0.86, 1.50)**
M01	Antiinflammatory and antirheumatic products	24.8	21.6	1.15 (0.86, 1.53)
**A**	**Alimentary tract and metabolism**	**24.8**	**14.0**	**1.78 (1.32, 2.39)** ^b^
A02	Drugs for acid related disorders	16.1	9.3	1.73 (1.18, 2.54) ^b^
A11	Vitamins	5.6	0.6	9.00 (3.71, 21.83) ^b^
**H**	**Systemic hormonal preparations, excl. Sexual hormones and insulins**	**16.8**	**12.7**	**1.32 (0.91, 1.90)** ^b^
H03	Thyroid therapy	12.4	9.4	1.32 (0.86, 2.05)
H02	Corticosteroids for systemic use	6.8	3.4	2.00 (1.07, 3.74) ^b^
**R**	**Respiratory system**	**15.5**	**15.1**	**1.03 (0.70, 1.50)**
**D**	**Dermatologicals**	**13.0**	**11.3**	**1.15 (0.76, 1.76)**
**C**	**Cardiovascular system**	**12.4**	**6.3**	**1.98 (1.26, 3.11)** ^b^
C03	Diuretics	3.1	0.3	10.00 (2.93, 34.18) ^b^
**G**	**Genitourinary system and sexual hormones**	**11.8**	**7.7**	**1.53 (0.97, 2.41)**
**B**	**Blood and blood forming organs**	**7.5**	**3.5**	**2.11 (1.15, 3.84)** ^b^
**L**	**Antineoplastic and immunomodulating agents**	**4.3**	**1.6**	**2.80 (1.23, 6.37)** ^b^

^a^Data are only shown for subcodes that were prescribed in > 10% of PKU patients or those that were significant vs the control population. ATC category totals may not add up because subcategories with < 5 patients in the PKU group have been excluded from the table, but may still count to the total for the ATC class of drug

^b^ATC codes that had a significant PR vs the control population

One letter ATC codes (e.g. Dermatologicals, Cardiovascular system) are shown in bold

#### Late-diagnosed adult PKU patients

All of the 50 most frequent comorbidities were present in > 10% of the adult late-diagnosed PKU patients (Table 7). The most frequent recorded ICD-10-GM codes were essential primary hypertension (Prevalence = 61.1%), disorders of lipoprotein metabolism and other lipidemias (Prevalence = 52.3%), and dorsalgia (Prevalence = 42.1%). Disorders of lipoprotein metabolism and other lipidemias were significantly more prevalent in the late-diagnosed PKU population vs controls (PR = 1.30; 95% CI 1.13–1.49).

**Table 7:** Comorbidity profile of late-diagnosed adult PKU patients in 2015 in Germany

ICD-10-GM code	Description	Late-diagnosed PKU patients (*n* = 216) %	Control group (*n* = 2,160) %	PR (95% CI)
I10	Essential (primary) hypertension	61.1	58.4	1.05 (0.94, 1.17)
E78	Disorders of lipoprotein metabolism and other lipidemias	52.3	40.4	1.30 (1.13, 1.49) ^a^
M54	Dorsalgia	42.1	36.1	1.17 (0.99, 1.38)
H52	Disorders of refraction and accommodation	38.0	33.1	1.15 (0.96, 1.37)
Z12	Encounter for screening for malignant neoplasms	28.2	30.0	0.94 (0.75, 1.18)
I25	Chronic ischemic heart disease	25.9	15.6	1.67 (1.30, 2.13) ^a^
E11	Type 2 diabetes mellitus	25.5	19.6	1.30 (1.02, 1.65) ^a^
Z25	Need for immunization against other single viral diseases	22.2	20.4	1.09 (0.84, 1.42)
M17	Osteoarthritis of knee	21.3	17.2	1.24 (0.94, 1.63)
Z00	Encounter for general exam without complaint, suspected or reported dx	20.8	20.2	1.03 (0.79, 1.36)
N95	Menopausal and other perimenopausal disorders	20.4	14.5	1.40 (1.06, 1.86) ^a^
E04	Other nontoxic goiter	19.9	14.7	1.35 (1.02, 1.80) ^a^
M47	Spondylosis	19.4	18.8	1.03 (0.78, 1.37)
E66	Overweight and obesity	19.0	14.2	1.34 (0.99, 1.79)
F32	Major depressive disorder, single episode	19.0	15.8	1.20 (0.90, 1.61)
M53	Other and unspecified dorsopathies, not elsewhere classified	19.0	12.6	1.51 (1.12, 2.03) ^a^
Z01	Encounter for other specified exam without complaint, suspected or reported dx	18.5	12.9	1.43 (1.06, 1.94) ^a^
E14	Unspecified diabetes mellitus	18.5	10.7	1.73 (1.28, 2.35) ^a^
E79	Disorders of purine and pyrimidine metabolism	17.6	11.1	1.59 (1.16, 2.17) ^a^
N39	Other disorders of urinary system	17.6	12.5	1.40 (1.03, 1.91) ^a^
Z92	Personal history of medical treatment	17.1	13.0	1.32 (0.97, 1.81)
F45	Somatoform disorders	17.1	14.3	1.20 (0.88, 1.64)
J44	Other chronic obstructive pulmonary disease	16.2	8.2	1.97 (1.41, 2.75) ^a^
J06	Acute upper respiratory infections of multiple and unspecified sites	15.7	12.2	1.29 (0.93, 1.79)
K29	Gastritis and duodenitis	15.7	11.1	1.42 (1.02, 1.98) ^a^
R42	Dizziness and giddiness	14.8	8.3	1.79 (1.26, 2.53) ^a^
I83	Varicose veins of lower extremities	14.8	13.0	1.14 (0.82, 1.60)
Z96	Presence of other functional implants	14.4	14.7	0.97 (0.69, 1.37)
N89	Other noninflammatory disorders of vagina	14.4	10.6	1.35 (0.96, 1.92)
M19	Other and unspecified osteoarthritis	14.4	10.1	1.42 (1.00, 2.01)
H35	Other retinal disorders	13.9	11.3	1.23 (0.86, 1.75)
L30	Other and unspecified dermatitis	13.9	9.0	1.54 (1.08, 2.20) ^a^
I70	Atherosclerosis	13.9	7.6	1.82 (1.26, 2.61) ^a^
J45	Asthma	13.4	6.9	1.93 (1.33, 2.81) ^a^
I50	Heart failure	13.4	8.3	1.61 (1.12, 2.32) ^a^
M21	Other acquired deformities of limbs	13.0	5.0	2.59 (1.75, 3.83) ^a^
E03	Other hypothyroidism	13.0	10.7	1.21 (0.84, 1.75)
N18	Chronic kidney disease	13.0	7.9	1.64 (1.13, 2.38) ^a^
N40	Enlarged prostate	13.0	12.3	1.06 (0.73, 1.52)
K76	Other diseases of liver	12.5	10.6	1.18 (0.82, 1.72)
M16	Osteoarthritis of hip	12.5	8.9	1.41 (0.96, 2.05)
H25	Age-related cataract	12.5	11.2	1.12 (0.77, 1.62)
D22	Melanocytic nevi	12.5	9.4	1.34 (0.92, 1.95)
I49	Other cardiac arrhythmias	12.0	10.0	1.20 (0.82, 1.76)
Z95	Presence of cardiac and vascular implants and grafts	12.0	8.1	1.48 (1.00, 2.18)
M51	Thoracic, thoracolumbar, and lumbosacral intervertebral disc disorders	11.6	12.8	0.91 (0.62, 1.33)
R10	Abdominal and pelvic pain	11.6	10.1	1.14 (0.77, 1.69)
M42	Spinal osteochondrosis	11.6	8.5	1.36 (0.92, 2.01)
H61	Other disorders of external ear	11.6	10.5	1.11 (0.75, 1.63)

^a^Comorbidities that had a significant PR vs the control population

Among the 50 most frequent comorbidities, those with a significant PR > 1.5 vs the matched control population are chronic ischemic heart disease (Prevalence = 25.9%; PR = 1.7; 95% CI 1.13–2.13), unspecified diabetes mellitus (Prevalence = 18.5%; PR = 1.7; 95% CI 1.28–2.35), disorders of purine and pyrimidine metabolism (Prevalence = 17.6%; PR = 1.6; 95% CI 1.16–2.17), other chronic obstructive pulmonary disease (Prevalence = 16.2%; PR = 2.0; 95% CI 1.41–2.75), dizziness and giddiness (Prevalence = 14.8%; PR = 1.8; 95% CI 1.26–2.53), atherosclerosis (Prevalence = 13.9%, PR = 1.8; 95% CI 1.26–2.61), asthma (Prevalence = 13.4%; PR = 1.9; 95% CI 1.33–2.81), heart failure (Prevalence = 13.4%; PR = 1.6; 95% CI 1.12–2.32), chronic kidney disease (Prevalence = 13.0%; PR = 1.6; 95% CI1.13–2.38), and other acquired deformities of limbs (Prevalence = 13.0%; PR = 2.6; 95% CI 1.75–3.83). A complete list of the 50 most prevalent comorbidities of the late-diagnosed PKU cohort and the corresponding prevalence in the control cohort is provided in Additional file 1: Table S7.

The most common ATC categories of prescribed agents that are significantly more prevalent in late-diagnosed PKU population vs controls (Table 8) are alimentary tract and metabolism (52.3% vs 41.3%; PR 1.27; 95% CI 1.10, 1.45), nervous system (50.5% vs 36.3%; PR 1.39; 95% CI 1.20, 1.60), and dermatologicals (28.7% vs 18.8%; PR 1.53; 95% CI 1.22, 1.92). The most common ATC subcodes for the prescribed agents with significant differences between the late-diagnosed and control populations are for beta-blockers (39.4% vs 32.6%; PR 1.21; 95% CI 1.01, 1.44), acid-related disorders (39.4% vs 28.3%; PR 1.39; 95% CI 1.16, 1.66), analgesics (33.3% vs 24.8%; PR 1.35; 95% CI 1.10, 1.65), and lipid-modifying agents (32.4% vs 22.5%; PR 1.44; 95% CI 1.17, 1.77). Additionally, diuretics, pyschoanaleptics, antigout preparations, corticosteroids for systemic use, corticosteroid dermatological preparations, antimycotics for dermatological use, drugs for obstructive airway diseases, and gynecological antiinfectives and antiseptics were all prescribed significantly more often in the late-diagnosed vs control populations. The 50 most frequently prescribed agents in late-diagnosed PKU patients are provided in full in Additional file 1: Table S8.

**Table 8:** ATC code and subcode of the top 50 most commonly prescribed agents in the late-diagnosed PKU population^a^

ATC code or subcode	Category or subcategory	Late-diagnosed PKU patients (*n* = 216) %	Control group (*n* = 2,161) %	PR (95% CI)
**C**	**Cardiovascular system**	**67.1**	**60.6**	**1.11 (1.00, 1.22)**
C09	Agents acting on the renin-angiotensin system	47.7	44.7	1.07 (0.92, 1.24)
C07	Beta blocking agents	39.4	32.6	1.21 (1.01, 1.44) ^b^
C10	Lipid modifying agents	32.4	22.5	1.44 (1.17, 1.77) ^b^
C03	Diuretics	25.5	17.9	1.42 (1.11, 1.82) ^b^
C08	Calcium channel blockers	19.9	15.1	1.32 (0.99, 1.76)
C01	Cardiac therapy	10.2	5.4	1.90 (1.23, 2.93) ^b^
**A**	**Alimentary tract and metabolism**	**52.3**	**41.3**	**1.27 (1.10, 1.45)** ^b^
A02	Drugs for acid related disorders	39.4	28.3	1.39 (1.16, 1.66) ^b^
A10	Antidiabetics	15.7	13.4	1.17 (0.85, 1.63)
**N**	**Nervous system**	**50.5**	**36.3**	**1.39 (1.20, 1.60)** ^b^
N02	Analgesics	33.3	24.8	1.35 (1.10, 1.65) ^b^
N06	Psychoanaleptics	19.4	12.0	1.62 (1.20, 2.17) ^b^
**M**	**Musculo-skeletal system**	**42.6**	**39.2**	**1.09 (0.92, 1.28)**
M01	Antiinflammatory and antirheumatic products	32.9	31.9	1.03 (0.84, 1.26)
M04	Antigout preparations	13.0	8.3	1.56 (1.08, 2.27) ^b^
M05	Drugs for treatment of bone diseases	3.7	2.6	1.43 (0.69, 2.96)
**J**	**Antiinfectives for systemic use**	**38.0**	**33.5**	**1.13 (0.95, 1.36)**
J01	Antibacterials for systemic use	36.1	32.0	1.13 (0.93, 1.36)
**H**	**Systemic hormonal preparations, excl. Sexual hormones and insulins**	**30.6**	**26.5**	**1.15 (0.93, 1.43)**
H03	Thyroid therapy	19.9	19.7	1.01 (0.76, 1.34)
H02	Corticosteroids for systemic use	13.0	9.4	1.37 (0.95, 1.99)
**D**	**Dermatologicals**	**28.7**	**18.8**	**1.53 (1.22, 1.92)** ^b^
D07	Corticosteroid, dermatological preparations	16.2	11.2	1.45 (1.05, 2.01) ^b^
D01	Antimycotics for dermatological use	7.9	4.7	1.68 (1.03, 2.76) ^b^
**R**	**Respiratory system**	**25.9**	**17.6**	**1.47 (1.15, 1.87)** ^b^
R03	Drugs for obstructive airway diseases	18.1	11.8	1.53 (1.13, 2.08) ^b^
**B**	**Blood and blood forming organs**	**22.7**	**22.6**	**1.00 (0.77, 1.30)**
B01	Antithrombotic agents	20.4	19.7	1.03 (0.78, 1.36)
**G**	**Genitourinary system and sexual hormones**	**21.3**	**14.4**	**1.47 (1.12, 1.94)** ^b^
G04	Urologicals	12.0	8.6	1.40 (0.95, 2.06)
G01	Gynecological antiinfectives and antiseptics	3.2	0.6	5.38 (2.17, 13.35) ^b^
**S**	**Sensory organs**	**10.6**	**14.4**	**0.74 (0.50, 1.11)**

^a^Data are only shown for subcodes that were prescribed in > 10% of PKU patients or those that were significant vs the control population. ATC category totals may not add up because subcategories with < 5 patients in the PKU group have been excluded from the table, but may still count to the total for the ATC class of drug

^b^ATC codes that had a significant PR vs the control population

One letter ATC codes (e.g. Dermatologicals, Cardiovascular system) are shown in bold

## Discussion

This study was designed to generate additional insights into the clinical burden of adult patients with PKU in Germany compared with the general population.

The unbiased design of this study, only selecting the 50 most common comorbidities and comedications in the PKU population and comparing with a rigorously matched control population, showed several surprising results. While the presence of neuropsychological conditions (e.g. depression and anxiety) at a higher prevalence in the PKU vs control population was to be expected in this analysis, the high prevalence of cardiovascular risk factors/conditions in the PKU population was unexpected. More than a third of adult PKU patients suffered from essential primary hypertension and disorders of lipoprotein metabolism and other lipidemias, while more than 10% had chronic ischemic heart disease, unspecified diabetes mellitus, or obesity. Furthermore, in all of these conditions, except primary hypertension, there was a significantly higher prevalence in the overall PKU population vs matched controls. It is worth noting that several of these conditions are components of metabolic syndrome [13].

The higher comorbid burden in PKU patients is also supported by the significantly higher proportion of patients with CCI scores ≥3 compared with the control population. Indeed, several comorbidities that contribute to the CCI score (e.g. diabetes mellitus, chronic kidney disease [CKD], chronic obstructive pulmonary disease) were found to be significantly more prevalent in the overall PKU population, early-diagnosed PKU population and late-diagnosed PKU population vs controls.

The observed difference in the prevalence of cardiovascular risk factors and diseases is reflected in the pattern of prescribed agents in this PKU population: 43.8% of the PKU population were receiving cardiovascular medicine vs 37.4% of the control population. Furthermore, beta-blockers, lipid-modifying agents, diuretics, cardiac therapy, and calcium-channel blockers were all prescribed significantly more often in the PKU vs control populations.

Treatments for acid-related disorders were prescribed in > 25% of PKU patients and at a significantly higher level than observed in matched controls, which may be due to the PKU diet.

Our study assessed a prevalence of adult PKU patients in 2015 in Germany (1 in 9,872) that is consistent with the reported prevalence/incidence of PKU among newborns of 1 in 6,000 to 1 in 10,000 live births [14, 15].

Although our analysis is unable to derive information on the degree of blood Phe control or disease severity exhibited by these patients, it is worth noting that < 1.3% of the overall PKU population (< 5 of the 377 PKU patients) were receiving sapropterin (all early-diagnosed patients) and only 13.8% of the overall PKU population were receiving D.A.S., again mainly in the early-diagnosed group (29.2% of early-diagnosed patients vs 2.6% of the late-diagnosed patients). This may indicate that relatively few of the late-diagnosed patients are well-controlled or on-diet vs the early-diagnosed patients.

When we consider the early-diagnosed population, they have a higher likelihood of their condition being continuously managed from an early age, they are relatively younger adults (mean age 30.7 years), and approximately 30% of them are receiving D.A.S. as part of their PKU management regime. Despite this, more than 10% of the population have an ICD code for conditions such as overweight and obesity (11.8%), other anxiety disorders (10.6%), and reaction to severe stress and adjustment disorders (13.7%). Furthermore, several conditions are significantly more prevalent in the early-diagnosed PKU population vs age-matched control subjects, including hypotension (PR 2.78), major depressive episodes (PR = 2.1), and disorders of lipid metabolism and other lipidemias (PR = 1.8).

The etiology of the comorbidities identified in this study cannot be ascertained from this type of study, but several interesting hypotheses can be generated based on knowledge of the underlying condition and the associated dietary management.

For instance, the higher level of risk for chronic ischemic heart disease in late-diagnosed PKU patients (Prevalence = 15.7%; PR = 1.7; 95% CI 1.30–2.13) could be associated with the higher prevalence of disorders of lipoprotein metabolism in this cohort (Additional file 1: Table S7) or several cardiometabolic anomalies that have been previously identified in PKU patients. Several published studies identify an increased or reduced risk of atherosclerosis or associated cardiovascular risk factors in PKU patients.

A recent study [16] demonstrated increased aortic stiffness in PKU patients (*n* = 41, 6 to 50 years of age), measured by applanation tonometry, when compared with a matched healthy control group and this was associated with higher Phe levels. However, another study [17] did not identify any difference in arterial stiffness or carotid intima media thickness (a surrogate marker of atherosclerosis) between PKU patients (*n* = 43, mean age 28.1 [SD 0.96]) and non-PKU control subjects (*n* = 58).

A correlation between elevated blood Phe levels and increased blood pressure has been demonstrated [18] in a study of 141 patients (6 months to 50 years of age) with classical PKU (*n* = 66; blood Phe ≥1200 μmol/L), mild-moderate PKU (*n* = 34; blood Phe 360–1200 μmol/L), or mild hyperphenylalaninemia (*n* = 41; MHPA; blood Phe 120–360 μmol/L). Patients with PKU (*n* = 100) had higher blood pressure than those with MHPA .

In contrast to the identified risk for ischemic heart diseases, lower levels of LDL cholesterol have been observed in adults with PKU, which may be simply due to the PKU diet or possibly via a direct effect of high blood Phe levels on cholesterol synthesis [19]. Another study [18] demonstrated that although total and LDL cholesterol were lower in classical PKU vs MHPA patients, lipid markers seemed to correlate with adherence to a PKU diet, as they were lower in treated PKU patients vs untreated or less stringently treated PKU patients. This may indicate that, regardless of the severity of PKU, lipid markers could be improved by adherence to diet. Of note, overweight or obese PKU patients in this study exhibited an atherogenic lipid profile (elevated levels of triglycerides, total cholesterol, LDL cholesterol and reduced levels of high-density lipoprotein [HDL] cholesterol), in addition to elevated levels of high sensitivity C-reactive protein (hsCRP).

Another study [20] in 59 patients with PKU and 44 healthy controls (11 to 17 years of age) found significantly lower levels of cardioprotective HDL cholesterol in well-controlled (*n* = 24; blood Phe < 360 μmol/L) vs poorly-controlled (*n* = 35; blood Phe > 360 μmol/L) PKU patients; both groups were significantly lower than non-PKU controls. Additionally, higher levels of homocysteine and increased mean platelet volume levels were also observed in PKU patients vs healthy controls and differences in these parameters were more evident in poorly-controlled PKU patients [20].

In summary, there is no consistent evidence that PKU patients may be at a higher risk for developing atherosclerosis. However, all of the cited studies were performed in relatively young PKU patients, and therefore, the effect of chronic, longer-term exposure to elevated blood Phe or the PKU diet could not be assessed. Our study provides a snapshot of the comorbidities present in an older population (late-diagnosed) of patients with PKU and demonstrated a significant PR vs controls for both risk factors (disorders of lipoprotein metabolism and other lipidemias) and cardiovascular disease (chronic ischemic heart disease and atherosclerosis). Further studies in older populations of PKU patients are required to confirm this association and elucidate the etiology.

An increased risk for being overweight or having obesity in dietary treated PKU patients, as found in early-diagnosed PKU patients in our study (Prevalence = 11.8; PR = 1.7; 95% CI 1.05–2.63), has been widely discussed in a review by Rocha et al. [21], although it could not be ascertained if weight issues were a result of the underlying condition (PKU), a consequence of treatment (PKU diet), or due to inadequate metabolic control. A study of 236 patients with PKU (mean age 26 [SD 7] years) proposed that an increased proportion of obese individuals may simply reflect the trends seen in the general population, but they did find a correlation between increasing body mass index (BMI) and higher blood Phe concentrations [22].

A study of BMI data from 947 patients with PKU (1.7 months to 26 years) found that in both children and adults with PKU (< 18 and > 19 years of age, respectively), females appear particularly vulnerable to excess weight gain and this may lead to a higher risk of atherosclerosis in PKU patients [23]. In our study, only early-diagnosed PKU patients showed a tendency to be overweight/obese compared with the control group. However, we do not know the proportion of PKU patients that were following a PKU diet or the degree of blood Phe control/lack of Phe control. However, we do know that approximately 98 and 70% of late- and early-diagnosed patients, respectively, were not receiving D.A.S.

In our study, both unspecified diabetes mellitus (Prevalence = 18.5; PR = 1.7; 95% CI 1.28–2.35) and type 2 diabetes mellitus (Prevalence 25.5; PR = 1.3: 95% CI 1.02–1.65) were more prevalent in late-diagnosed PKU patients vs control subjects. In addition to being a serious chronic condition, diabetes is also a significant risk factor for both cardiovascular and renal disease. Given these findings, the management of these patients may need to include assessment of insulin levels and the Homeostatic Model Assessment of Insulin Resistance (HOMA-IR) index.

Because of the high carbohydrate intake inherent in the PKU diet, there has been copious discussion regarding an increased risk of diabetes in these patients. However, there is currently no clear evidence that patients with PKU exhibit a higher risk of developing diabetes and most studies only include children or young adults, which may exclude the development timeline of type 2 diabetes mellitus.

It is interesting to note that several of the conditions identified among PKU patients in this study (diabetes mellitus, dyslipidemia, obesity) are constituents of the metabolic syndrome. Kanufre et al. [24] found that overweight PKU patients may be vulnerable to the development of the metabolic syndrome.

Our study includes patients aged 18–92 years and therefore includes older age groups, especially in the late-diagnosed population (range 46–96 years), that are not represented in published studies addressing cardiometabolic comorbidities in adults with PKU. Studies are required assessing the long-term effect of various atherogenic factors like obesity, diabetes mellitus, hypertension, oxidative stress and other factors which may not be evident in younger patient populations. Many of the late-diagnosed PKU patients (median age 65 years in our study) may be in institutions or nursing homes. It is well known that patients living in institutions have a lower life expectancy [25]. Findings indicate that the mean prevalence of heart failure is 20% (range 15–45%) and that there is a significant level of comorbidity (dementia, diabetes mellitus, and chronic obstructive pulmonary disease) in nursing home residents with heart failure [26, 27].

The finding that late-diagnosed PKU patients exhibit a higher prevalence of CKD compared with their matched controls (Prevalence = 13.0%; PR = 1.6; 95% CI 1.13–2.38) is an interesting finding and there is evidence to suggest that the PKU diet also may be a factor. In a well-controlled study analyzing renal function in 67 patients with PKU, Hennermann et al. [28] demonstrated that 19% of PKU patients had impaired renal function, 31% had proteinuria, and 23% had arterial hypertension. Furthermore, renal function declined with increasing protein intake. The authors propose a negative impact of amino acid supplementation on renal function, but additional studies are required to confirm these findings.

There is a plethora of evidence supporting the role of oxidative stress as an underlying factor in the etiology of several diseases, including atherosclerosis, chronic kidney disease, and diabetes (for review see Liguori et al. [29]). The evidence for increased levels of oxidative stress in PKU patients and the role it plays in PKU has been previously discussed [30, 31].

Preissler et al. [32] found that oxidative stress is induced in cultured astrocytes by concentrations of Phe normally found in PKU patients and that this may lead to cell death. Two studies have found evidence of increased oxidative stress in PKU patients [33, 34] that was associated with increased levels of DNA or tissue damage, even in well-controlled PKU patients. In summary, increased oxidative stress in PKU patients is evident; however, there are no rigorous studies investigating if this translates into a higher risk of atherosclerosis or other diseases in PKU patients.

The results of a similar study were recently published by Burton et al. [35]; the identified comorbidities among PKU patients in the US show some similarities to those present in the German PKU patients. Although a direct comparison of the two populations may be limited - e.g. the study compared the prevalence of comorbidities selected by an expert panel of physicians (rather than the most prevalent comorbidities), used the ICD-9 coding (rather than ICD-10) and the US population was made up of younger patients (mean age approximately 35 years), mostly born after the start of NBS - similar PR’s were found for several comorbidities including overweight and obesity, gastrointestinal disorders, and asthma. One may speculate that this is due to the Phe-restricted diet featuring high amounts of amino acid supplements, which may contribute to the presence of oesophagitis and gastroesophageal reflux. On the other hand, an increased prevalence of cardiovascular diseases was not found in the US investigation, which is likely due to the lower age of patients. In the US study, renal insufficiency (both with and without hypertension) and calculus of the kidney were identified as significantly more prevalent in the PKU vs control population. Although our study identified a significant PR for CKD in the late-diagnosed population vs controls, renal insufficiency and renal complications were not among the top 50 comorbidities in the early-diagnosed population, who are more comparable to the US study population (average age 31 vs 35 years). This may be due to the different approaches to treatment; only 2.6% of the late-diagnosed group and 29.2% of the early-diagnosed group in Germany are prescribed amino acid supplements. In the study by Hennermann et al. [28], it was hypothesized that renal excretion of amino acids may be responsible for renal damage. It is also of note that several comorbidities that may be amenable to prevention (e.g. obesity, hypertension, dyslipidemia) are more prevalent in the early-treated population vs their controls, as well as in the late-treated population. While this may be expected in the late-treated population, the presence of these comorbidities in the early-treated population may reflect the focus of care (i.e. control of blood Phe and diet) in patients with PKU and that assessment/management of these comorbidities may need to become part of clinical practice.

### Strengths and limitations

Claims data analyses are primarily collected for reimbursement purposes and do not necessary cover clinical parameters. Therefore, the study had to rely on the information that is coded in the ICD-10-GM catalog. The ICD-10-GM catalog provides information about the disorders of aromatic amino-acid metabolism, but contains no specific codes for the severity of PKU. Therefore, we may have included patients with a very mild form of PKU, which could result in underestimating the burden of disease for the severe PKU patients.

PKU patients might be screened more frequently, due to their annual (or more frequent) visits to their PKU clinic, leading to a higher rate of detection of comorbidities vs control subjects.

The higher proportion of females in the PKU group, especially in the early-diagnosed population, could be due to the recommendation that females of reproductive age be screened for risks associated with maternal PKU [4].

The stratification of the study population into early-diagnosed and late-diagnosed PKU patients was based exclusively on the year of birth in relation to the implementation of NBS for PKU in Germany during 1969/1970. This approach does not account for patients who were born in 1969/1970 (who may or not have been screened at birth), patients who may have been born in other countries [36], or for patients born before 1969 with older siblings with diagnosed PKU (who were therefore diagnosed at birth).

On the other hand, this study has some major strengths. First, the utilized data source allows generalization of our results to a major part of the German population, as approximately 85% of the German population are covered by statutory health insurance (SHI). In contrast to registries and clinical trials, where a selected population is investigated, this analysis should not be affected by a selection bias. Also, participants of the German SHI system benefit from nearly full coverage of all healthcare services; minor copayments exist but these are limited to 2% of the annual income of the insured individuals (1% for chronically ill individuals). German claims data therefore provides a near-complete picture of all direct healthcare utilization; therefore, our study should provide a complete picture of comorbidities and any prescribed medications.

### Generalizability

The InGef research database is based on claims data from the SHI system, but is adjusted to the German overall population in terms of age and sex. As proportionately more males choose private health insurance in Germany, the proportion of females is higher in the SHI population than in the overall German population; this limits the generalizability of our results. On the other hand, the generalizability of the results to the German population might be biased because individuals with an annual income above a defined threshold could choose a private health insurance instead of the SHI. These individuals tend to be healthier than the individuals that have to be insured by the SHI [37]. Moreover, the prevalence of PKU shows regional differences among the federal states in Germany. The adjusted age and sex distribution of the InGef research database does not account for these regional differences [38].

## Conclusions

This retrospective matched cohort analysis using German SHI claims data assessed the clinical burden of PKU in Germany. Adult PKU patients, even those who are early-diagnosed, suffer not only from the direct burden of PKU, but are also likely to present with additional comorbidities, including cardiometabolic risk factors, that impact patients’ lives. An increased healthcare burden is reflected by a higher intake of prescriptions of gastrointestinal agents, analgesics and antipyretics, statins, and antidepressants. The matched comparison revealed that PKU patients suffered more often from intellectual, developmental, and psychological disorders and that PKU patients, especially those who are late-diagnosed, have a higher burden of disease compared with the general population. Future studies in adult PKU patients must clarify if these comorbidities, several of which were not expected in this population, are caused by environmental conditions, the underlying disease, or are related to the requisite treatment.

## Methods

### Study design

This study was designed as a retrospective matched cohort analysis comparing PKU patients with matched controls from the general population. The study utilized German statutory health insurance (SHI) claims data and was conducted from the perspective of the German SHI.

### Data source

The Institut für angewandte Gesundheitsforschung Berlin (InGef) research database contains anonymized healthcare claims of approximately four million covered lives. It is adjusted to the overall German population in terms of age and gender and is considered to be in good accordance to the overall German population for measures of morbidity, mortality, and drug usage [39]. The InGef research database includes a geographically well-distributed population from all federal states of Germany, which is insured by approximately 70 different insurance companies. The claims data are regularly audited by the insurance companies for reimbursement purposes and are prepared in accordance with German Social Law (paragraphs 287 SGB V and 75 SGB X). Data on patients and physicians is anonymized, as are the providers and the health insurances, before data is made available to the InGef, ensuring compliance with the strict data protection regulations in Germany.

### Study period

The study period was from January 1, 2010 to December 31, 2015. PKU patients were enrolled within this time frame (enrollment period) and the outcomes were analyzed for a 1-year period from January 1, 2015 to December 31, 2015 (outcomes observation period).

### Study population

PKU patients were identified using International Statistical Classification of Diseases and Related Health Problems, 10th revision, German Modification (ICD-10-GM) codes (E70.0 [Classical phenylketonuria] or E70.1 [Other hyperphenylalaninemias]) in the inpatient (main or secondary discharge diagnoses) and/or outpatient setting (verified diagnoses) during the enrollment period. They were excluded if they were younger than 18 years of age on January 1, 2015 or if they were lost to follow-up due to a sickness fund switch within the outcomes observation period.

### Subgroups

The adult PKU cohort was divided into early-diagnosed and late-diagnosed patients based on their birth year in relation to the implementation of newborn screening (NBS) for PKU in Germany between 1969 and 1970 [40]. Hence, adult PKU patients born prior to 1969 were presumed to be late-diagnosed.

### Matching

For each of the eligible adult PKU patients, ten controls were drawn from the InGef research database via direct, exact matching, without replacement on age and sex. Non-PKU controls (no PKU diagnosis code in the enrollment period) were required to be continuously enrolled in the database during the outcomes observation period, except for patients who died.

Matching balance was measured by the standardized difference with a threshold of 10%, indicating an imbalance of the matching parameters if the standardized difference exceeds the threshold [41–44]*.*

### Outcomes

The 50 most common comorbidities among the overall adult PKU cohort, the early-diagnosed PKU cohort, and the late-diagnosed PKU cohort in 2015 were identified and rank-ordered using ICD-10-GM codes and the prevalence of those comorbidities compared with the matched control group. The most commonly prescribed concomitant medications in 2015 were identified using 7-digit Anatomical Therapeutic Chemical (ATC) codes and pharmaceutical central numbers (PZN) and prescribing levels compared with the matched control group. Differences between the groups were tested using 95% confidence intervals (95% CI) of prevalence ratio (PR).

Additionally, the Updated Charlson Comorbidity Index (CCI) was analyzed to measure the overall health status [45]*.* The CCI is a weighted index that takes into account the number and the seriousness of comorbid diseases by assigning points for certain illnesses. The CCI score is the sum of the points for each disease and higher scores indicate a greater burden of disease; scores run from 0 to 29, but are generally presented categorically as 0, 1, 2, 3 and ≥ 4. Component comorbidities of the CCI are myocardial infarction, congestive heart failure, peripheral vascular disease, cerebrovascular disease, hemiplegia or paraplegia, dementia, chronic pulmonary disease, rheumatologic disease, peptic ulcer disease, diabetes without chronic complications, diabetes with chronic complications, renal disease, any malignancy (including leukemia and lymphoma), metastatic solid tumor, mild liver disease, moderate or severe liver disease, and acquired immune deficiency syndrome (AIDS)/human immunodeficiency virus (HIV). Differences between groups for CCI scores were tested using a chi-square test and 95% CI of PR.

## Additional file

Additional file 1: Tables S1-S8. (DOCX 86 kb)

## Data Availability

The utilized database in this study is available from the Intitut für angewandte Gesundheitsforschung Berlin (InGef) but restrictions apply to the availability of these data, which were used under license for the current study, and so are not publicly available.
