# Supplementary material for: Clinical burden of illness in patients with phenylketonuria (PKU) and associated comorbidities - a retrospective study of German health insurance claims data
Source: Orphanet J Rare Dis. 2019 Jul 22;14:181. doi: 10.1186/s13023-019-1153-y (PMC6647060; doi:10.1186/s13023-019-1153-y)
Supplement: Supplementary file 1 — Tables S1-S8. (DOCX 86 kb) [file 13023_2019_1153_MOESM1_ESM.docx]

**Additional file 1**

**Table S1.** CCI categories among the late-diagnosed PKU cohort and the matched cohort

|  | Late-diagnosed PKU cohort (n=216) | | Control cohort (n=2,160) | | PR (95% CI) |
| --- | --- | --- | --- | --- | --- |
|  | **n** | **%** | **n** | **%** |  |
| CCI=0 | 71 | 32.9 | 906 | 41.9 | 0.78 (0.64, 0.95) |
| CCI=1 | 42 | 19.4 | 473 | 21.9 | 0.89 (0.67, 1.18) |
| CCI=2 | 30 | 13.9 | 302 | 14.0 | 0.99 (0.70, 1.41) |
| CCI=3 | 28 | 13.0 | 177 | 8.3 | 1.58 (1.09, 2.30)**^†^** |
| CCI≥4 | 45 | 20.8 | 302 | 14.0 | 1.49 (1.13, 1.97)**^†^** |

^†^statistically significant

**Table S2.** CCI categories among the early-diagnosed PKU cohort and the matched cohort

|  | Early-diagnosed PKU cohort (n=161) | | Control cohort (n=1,610) | | PR (95% CI) |
| --- | --- | --- | --- | --- | --- |
|  | **n** | **%** | **n** | **%** |  |
| CCI=0 | 123 | 76.4 | 1,300 | 80.7 | 0.95 (0.87, 1.03) |
| CCI=1 | 30 | 18.6 | 243 | 15.1 | 1.23 (0.88, 1.74) |
| CCI=2 | 5 | 3.1 | 51 | 3.2 | 0.98 (0.40, 2.42) |
| CCI=3 | <5 | N/A | ≥5 | N/A | - |
| CCI≥4 | <5 | N/A | <5 | N/A | - |

**Table S3.** Top 50 most prevalent comorbidities of the PKU cohort and the corresponding prevalence in the control cohort in Germany in 2015

| ICD-10-GM code | Description | PKU cohort (n=377) | | Control cohort (n=3,770) | | PR (95% CI) |
| --- | --- | --- | --- | --- | --- | --- |
|  |  | **n** | **%** | **n** | **%** |  |
| I10 | Essential primary hypertension | 146 | 38.7 | 1,364 | 36.2 | 1.07 (0.94, 1.22) |
| M54 | Dorsalgia | 133 | 35.3 | 1,144 | 30.3 | 1.16 (1.01, 1.34)**^†^** |
| E78 | Disorders of lipoprotein metabolism and other lipidemias | 127 | 33.7 | 948 | 25.1 | 1.34 (1.15, 1.56)**^†^** |
| Z12 | Encounter for screening for malignant neoplasms | 118 | 31.3 | 1,131 | 30.0 | 1.04 (0.89, 1.22) |
| H52 | Disorders of refraction and accommodation | 103 | 27.3 | 889 | 23.6 | 1.16 (0.97, 1.38) |
| Z30 | Encounter for contraceptive management | 85 | 22.5 | 799 | 21.2 | 1.06 (0.87, 1.30) |
| N89 | Other noninflammatory disorders of vagina | 82 | 21.8 | 677 | 18.0 | 1.21 (0.99, 1.48) |
| J06 | Acute upper respiratory infections of multiple and unspecified sites | 81 | 21.5 | 708 | 18.8 | 1.14 (0.93, 1.40) |
| Z01 | Encounter for other specified exam without complaint, suspected or reported dx | 78 | 20.7 | 529 | 14.0 | 1.47 (1.19, 1.82)**^†^** |
| F32 | Major depressive disorder, single episode | 61 | 16.2 | 501 | 13.3 | 1.22 (0.95, 1.55) |
| E66 | Overweight and obesity | 60 | 15.9 | 421 | 11.2 | 1.43 (1.11, 1.83)**^†^** |
| Z00 | Encounter for general exam without complaint, suspected or reported dx | 60 | 15.9 | 541 | 14.4 | 1.11 (0.87, 1.42) |
| I25 | Chronic ischemic heart disease | 59 | 15.7 | 339 | 9.0 | 1.74 (1.35, 2.25)**^†^** |
| F45 | Somatoform disorders | 58 | 15.4 | 458 | 12.1 | 1.27 (0.98, 1.63) |
| E11 | Type 2 diabetes mellitus | 56 | 14.9 | 436 | 11.6 | 1.28 (0.99, 1.66) |
| M53 | Other and unspecified dorsopathies, not elsewhere classified | 54 | 14.3 | 385 | 10.2 | 1.40 (1.08, 1.83)**^†^** |
| Z25 | Need for immunization against other single viral diseases | 54 | 14.3 | 495 | 13.1 | 1.09 (0.84, 1.42) |
| E04 | Other nontoxic goiter | 53 | 14.1 | 408 | 10.8 | 1.30 (1.00, 1.69) |
| M47 | Spondylosis | 52 | 13.8 | 461 | 12.2 | 1.13 (0.86, 1.47) |
| M17 | Osteoarthritis of knee | 50 | 13.3 | 381 | 10.1 | 1.31 (1.00, 1.73) |
| R10 | Abdominal and pelvic pain | 50 | 13.3 | 429 | 11.4 | 1.17 (0.89, 1.53) |
| N39 | Other disorders of urinary system | 49 | 13.0 | 356 | 9.4 | 1.38 (1.04, 1.82)**^†^** |
| K29 | Gastritis and duodenitis | 48 | 12.7 | 364 | 9.7 | 1.32 (0.99, 1.75) |
| N95 | Menopausal and other perimenopausal disorders | 47 | 12.5 | 323 | 8.6 | 1.46 (1.09, 1.94)**^†^** |
| J45 | Asthma | 45 | 11.9 | 265 | 7.0 | 1.70 (1.26, 2.29)**^†^** |
| J30 | Vasomotor and allergic rhinitis | 44 | 11.7 | 333 | 8.8 | 1.32 (0.98, 1.78) |
| E03 | Other hypothyroidism | 44 | 11.7 | 357 | 9.5 | 1.23 (0.92, 1.66) |
| D22 | Melanocytic nevi | 44 | 11.7 | 345 | 9.2 | 1.28 (0.95, 1.71) |
| R42 | Dizziness and giddiness | 42 | 11.1 | 228 | 6.0 | 1.84 (1.35, 2.52)**^†^** |
| F43 | Reaction to severe stress, and adjustment disorders | 41 | 10.9 | 262 | 7.0 | 1.56 (1.15, 2.14)**^†^** |
| E14 | Unspecified diabetes mellitus | 41 | 10.9 | 243 | 6.4 | 1.69 (1.23, 2.31)**^†^** |
| Z92 | Personal history of medical treatment | 40 | 10.6 | 290 | 7.7 | 1.38 (1.01, 1.89)**^†^** |
| M79 | Other and unspecified soft tissue disorders, not elsewhere classified | 40 | 10.6 | 270 | 7.2 | 1.48 (1.08, 2.03)**^†^** |
| A09 | Infectious gastroenteritis and colitis, unspecified | 40 | 10.6 | 236 | 6.3 | 1.69 (1.23, 2.33)**^†^** |
| L30 | Other and unspecified dermatitis | 40 | 10.6 | 277 | 7.3 | 1.44 (1.05, 1.98)**^†^** |
| Q66 | Congenital deformities of feet | 39 | 10.3 | 303 | 8.0 | 1.29 (0.94, 1.77) |
| I83 | Varicose veins of lower extremities | 39 | 10.3 | 330 | 8.8 | 1.18 (0.86, 1.62) |
| E79 | Disorders of purine and pyrimidine metabolism | 38 | 10.1 | 255 | 6.8 | 1.49 (1.08, 2.06)**^†^** |
| T78 | Adverse effects, not elsewhere classified | 38 | 10.1 | 222 | 5.9 | 1.71 (1.23, 2.37)**^†^** |
| M51 | Thoracic, thoracolumbar, and lumbosacral intervertebral disc disorders | 37 | 9.8 | 331 | 8.8 | 1.12 (0.81, 1.54) |
| M21 | Other acquired deformities of limbs | 37 | 9.8 | 165 | 4.4 | 2.24 (1.60, 3.15)**^†^** |
| M25 | Other joint disorder, not elsewhere classified | 36 | 9.5 | 293 | 7.8 | 1.23 (0.88, 1.71) |
| J44 | Other chronic obstructive pulmonary disease | 36 | 9.5 | 191 | 5.1 | 1.88 (1.34, 2.65)**^†^** |
| N94 | Pain and other conditions associated with female genital organs and menstrual cycle | 36 | 9.5 | 246 | 6.5 | 1.46 (1.05, 2.04)**^†^** |
| F41 | Other anxiety disorders | 35 | 9.3 | 193 | 5.1 | 1.81 (1.29, 2.56)**^†^** |
| M99 | Biomechanical lesions, not elsewhere classified | 35 | 9.3 | 383 | 10.2 | 0.91 (0.66, 1.27) |
| M77 | Other enthesopathies | 34 | 9.0 | 233 | 6.2 | 1.46 (1.03, 2.06)**^†^** |
| F33 | Major depressive disorder, recurrent | 33 | 8.8 | 146 | 3.9 | 2.26 (1.57, 3.25)**^†^** |
| M19 | Other and unspecified osteoarthritis | 33 | 8.8 | 237 | 6.3 | 1.39 (0.98, 1.97) |

^†^statistically significant

**Table S4.** ATC code and subcode of the most commonly prescribed agents in the PKU cohort

| ATC code or subcode | Category or subcategory | PKU cohort (n=377)  % | Control cohort (n=3,770)  % | PR (95% CI) |
| --- | --- | --- | --- | --- |
| **C** | **Cardiovascular system** | **43.8** | **37.4** | **1.17 (1.04, 1.32)^†^** |
| C09 | Agents acting on the renin-angiotensin system | 30.0 | 27.0 | 1.11 (0.94, 1.31) |
| C07 | Beta blocking agents | 24.9 | 19.9 | 1.25 (1.04, 1.51)**^†^** |
| C10 | Lipid modifying agents | 19.4 | 13.1 | 1.48 (1.19, 1.85)**^†^** |
| C03 | Diuretics | 15.9 | 10.4 | 1.53 (1.19, 1.97)**^†^** |
| C08 | Calcium channel blockers | 12.5 | 8.9 | 1.40 (1.05, 1.86)**^†^** |
| C01 | Cardiac therapy | 6.1 | 3.1 | 1.95 (1.26, 3.01)**^†^** |
| C02 | Antihypertensives | 1.9 | 1.9 | 0.99 (0.46, 2.13) |
| C05 | Vasoprotectives | <5 | 0.7 | N/A |
| C04 | Peripheral vasodilators | <5 | <5 | N/A |
| **A** | **Alimentary tract and metabolism** | **40.6** | **29.6** | **1.37 (1.20, 1.56)^†^** |
| A02 | Drugs for acid related disorders | 29.4 | 20.2 | 1.46 (1.23, 1.72)**^†^** |
| A10 | Antidiabetics | 9.0 | 8.2 | 1.10 (0.79, 1.55) |
| A11 | Vitamines | 4.5 | 2.0 | 2.27 (1.35, 3.80)**^†^** |
| A12 | Minerals | 3.7 | 1.3 | 2.86 (1.59, 5.13)**^†^** |
| A03 | Drugs for functional gastrointestinal disorders | 3.2 | 3.7 | 0.86 (0.48, 1.54) |
| A01 | Stomatological preparations | 2.1 | 1.1 | 1.86 (0.88, 3.93) |
| A06 | Drugs for constipation | 1.9 | 1.8 | 1.03 (0.48, 2.22) |
| A07 | Antidiarrheals, intestinal antiinflammatory/ antiinfective agents | 1.6 | 1.1 | 1.50 (0.64, 3.51) |
| A04 | Antiemetics and antinauseants | <5 | 0.5 | N/A |
| A16 | Other alimentary tract and metabolism products | <5 | 0.0 | N/A |
| A09 | Digestives, incl. enzymes | <5 | 0.2 | N/A |
| **N** | **Nervous system** | **40.3** | **28.4** | **1.42 (1.24, 1.62)^†^** |
| N02 | Analgesics | 24.4 | 19.0 | 1.28 (1.06, 1.55)**^†^** |
| N06 | Psychoanaleptics | 17.2 | 9.5 | 1.82 (1.43, 2.31)**^†^** |
| N05 | Psycholeptics | 8.8 | 5.4 | 1.62 (1.14, 2.30)**^†^** |
| N03 | Antiepileptics | 5.3 | 2.9 | 1.82 (1.14, 2.89)**^†^** |
| N07 | Other nervous system drugs | 2.7 | 1.3 | 2.08 (1.06, 4.08)**^†^** |
| N04 | Anti-parkinson drugs | 2.1 | 1.1 | 1.86 (0.88, 3.93) |
| N01 | Anesthetics | <5 | 0.1 | N/A |
| **J** | **Antiinfectives for systemic use** | **36.6** | **34.0** | **1.08 (0.94, 1.24)** |
| J01 | Antibacterials for systemic use | 34.7 | 32.8 | 1.06 (0.92, 1.23) |
| J07 | Vaccines | 1.9 | 0.3 | 6.36 (2.48, 16.32)**^†^** |
| J05 | Antivirals for systemic use | 1.6 | 1.4 | 1.18 (0.51, 2.72) |
| J06 | Immune sera and immunoglobulins | <5 | 0.5 | N/A |
| J02 | Antimycotics for systemic use | <5 | 0.1 | N/A |
| **M** | **Musculo-skeletal system** | **35.3** | **32.0** | **1.10 (0.95, 1.27)** |
| M01 | Antiinflammatory and antirheumatic products | 29.4 | 27.5 | 1.07 (0.91, 1.26) |
| M04 | Antigout preparations | 7.7 | 4.9 | 1.58 (1.09, 2.31)**^†^** |
| M05 | Drugs for treatment of bone diseases | 2.4 | 1.5 | 1.61 (0.80, 3.22) |
| M03 | Muscle relaxants | 1.9 | 1.9 | 1.00 (0.46, 2.16) |
| **H** | **Systemic hormonal preparations, excl. sexual hormones and insulins** | **24.7** | **20.6** | **1.20 (0.99, 1.44)** |
| H03 | Thyroid therapy | 16.7 | 15.3 | 1.09 (0.86, 1.39) |
| H02 | Corticosteroids for systemic use | 10.3 | 6.9 | 1.51 (1.09, 2.07)**^†^** |
| H01 | Pituitary and hypothalamic hormones and analogues | <5 | 0.1 | N/A |
| H04 | Pancreatic hormones | <5 | 0.2 | N/A |
| H05 | Calcium homeostasis | <5 | <5 | N/A |
| **D** | **Dermatologicals** | **22.0** | **15.6** | **1.41 (1.15, 1.73)^†^** |
| D07 | Corticosteroide, dermatological preparations | 12.5 | 9.0 | 1.39 (1.04, 1.85)**^†^** |
| D01 | Antimycotics for dermatological use | 5.6 | 3.5 | 1.58 (1.01, 2.47)**^†^** |
| D06 | Antibiotics and chemotherapeutics for dermatological use | 3.4 | 2.3 | 1.51 (0.85, 2.68) |
| D11 | Other dermatological preparations | 1.6 | 1.1 | 1.46 (0.63, 3.42) |
| D02 | Emollients and skin protectives | <5 | 0.2 | N/A |
| D03 | Preparations for treatment of wounds and ulcers | <5 | <5 | N/A |
| D05 | Antipsoriatics | <5 | 0.6 | N/A |
| D08 | Antiseptics and disinfectants | <5 | 0.3 | N/A |
| D09 | Medical bandages | <5 | 0.6 | N/A |
| D10 | Antiacne preparations | <5 | 1.1 | N/A |
| **R** | **Respiratory system** | **21.5** | **16.6** | **1.30 (1.06, 1.59)^†^** |
| R03 | Drugs for obstructive airway diseases | 13.0 | 9.8 | 1.32 (1.00, 1.74) |
| R01 | Rhinologics | 5.8 | 4.1 | 1.42 (0.92, 2.19) |
| R05 | Cough and cold preparations | 5.0 | 3.3 | 1.54 (0.96, 2.47) |
| R06 | Antihistamines for systemic use | 3.2 | 2.3 | 1.36 (0.75, 2.47) |
| **G** | **Genitourinary system and sexual hormones** | **17.2** | **11.6** | **1.49 (1.17, 1.89)^†^** |
| G03 | Sexual hormones and modulators of the genital system | 8.0 | 5.7 | 1.40 (0.97, 2.01) |
| G04 | Urologicals | 7.4 | 5.1 | 1.44 (0.99, 2.11) |
| G01 | Gynecological antiinfectives and antiseptics | 3.2 | 1.4 | 2.31 (1.24, 4.28)**^†^** |
| G02 | Other gynecologicals | <5 | <5 | N/A |
| **B** | **Blood and blood forming organs** | **16.2** | **14.5** | **1.12 (0.88, 1.42)** |
| B01 | Antithrombotic agents | 13.3 | 12.0 | 1.10 (0.84, 1.45) |
| B03 | Antianemic preparations | 3.4 | 2.9 | 1.17 (0.67, 2.06) |
| B05 | Blood substitutes and perfusion solutions | 1.6 | 1.0 | 1.67 (0.71, 3.93) |
| **S** | **Sensory organs** | **8.8** | **11.4** | **0.77 (0.55, 1.08)** |
| S01 | Ophthalmologicals | 7.2 | 10.2 | 0.70 (0.48, 1.02) |
| S02 | Otologicals | 1.6 | 1.1 | 1.43 (0.61, 3.34) |
| **V** | **Various** | **6.6** | **4.6** | **1.45 (0.97, 2.18)** |
| V04 | Diagnostic agents | 5.0 | 3.7 | 1.38 (0.86, 2.20) |
| V01 | Allergens | <5 | 0.4 | N/A |
| V03 | All other therapeutic products | <5 | 0.3 | N/A |
| V07 | All other non-therapeutic products | <5 | 0.3 | N/A |
| V08 | Contrast media | <5 | <5 | N/A |
| **L** | **Antineoplastic and immunomodulating agents** | **4.5** | **3.5** | **1.30 (0.79, 2.13)** |
| L01 | Antineoplastic agents | 2.4 | 1.5 | 1.58 (0.79, 3.16) |
| L04 | Immunosuppressants | 1.6 | 0.9 | 1.71 (0.73, 4.05) |
| L02 | Endocrine therapy | <5 | 1.3 | N/A |
| L03 | Immunostimulants | <5 | 0.2 | N/A |
| **P** | **Antiparasitic products, insecticides and repellents** | **<5** | **1.6** | **N/A** |
| P01 | Antiprotozoals | <5 | 1.2 | N/A |
| P03 | Ectoparasiticides, incl. scabicides, insecticides and repellents | <5 | <5 | N/A |

^†^statistically significant

**Table S5.** Top 50 most prevalent comorbidities of the early-diagnosed PKU cohort and the corresponding prevalence in the control cohort in Germany in 2015

| ICD-10-GM code | Description | Early-diagnosed  PKU cohort (n=161) | | Control cohort (n=1,610) | | PR (95% CI) |
| --- | --- | --- | --- | --- | --- | --- |
|  |  | **n** | **%** | **n** | **%** |  |
| Z30 | Encounter for contraceptive management | 75 | 46.6 | 682 | 42.4 | 1.10 (0.92, 1.31) |
| Z12 | Encounter for screening for malignant neoplasms | 57 | 35.4 | 484 | 30.1 | 1.18 (0.94, 1.47) |
| N89 | Other noninflammatory disorders of vagina | 51 | 31.7 | 448 | 27.8 | 1.14 (0.90, 1.45) |
| J06 | Acute upper respiratory infections of multiple and unspecified sites | 47 | 29.2 | 444 | 27.6 | 1.06 (0.82, 1.36) |
| M54 | Dorsalgia | 42 | 26.1 | 364 | 22.6 | 1.15 (0.88, 1.52) |
| Z01 | Encounter for other specified exam without complaint, suspected or reported dx | 38 | 23.6 | 250 | 15.5 | 1.52 (1.13, 2.05)**^†^** |
| N94 | Pain and other conditions associated with female genital organs and menstrual cycle | 29 | 18.0 | 208 | 12.9 | 1.39 (0.98, 1.98) |
| R10 | Abdominal and pelvic pain | 25 | 15.5 | 210 | 13.0 | 1.19 (0.81, 1.74) |
| J30 | Vasomotor and allergic rhinitis | 24 | 14.9 | 184 | 11.4 | 1.30 (0.88, 1.93) |
| A09 | Infectious gastroenteritis and colitis, unspecified | 24 | 14.9 | 159 | 9.9 | 1.51 (1.01, 2.25)**^†^** |
| N92 | Excessive, frequent and irregular menstruation | 23 | 14.3 | 172 | 10.7 | 1.34 (0.89, 2.00) |
| F43 | Reaction to severe stress, and adjustment disorders | 22 | 13.7 | 126 | 7.8 | 1.75 (1.14, 2.67)**^†^** |
| H52 | Disorders of refraction and accommodation | 21 | 13.0 | 174 | 10.8 | 1.21 (0.79, 1.84) |
| F45 | Somatoform disorders | 21 | 13.0 | 150 | 9.3 | 1.40 (0.91, 2.15) |
| F32 | Major depressive disorder, single episode | 20 | 12.4 | 160 | 9.9 | 1.25 (0.81, 1.93) |
| E66 | Overweight and obesity | 19 | 11.8 | 114 | 7.1 | 1.67 (1.05, 2.63)**^†^** |
| M79 | Other and unspecified soft tissue disorders, not elsewhere classified | 18 | 11.2 | 88 | 5.5 | 2.05 (1.27, 3.31)**^†^** |
| F41 | Other anxiety disorders | 17 | 10.6 | 85 | 5.3 | 2.00 (1.22, 3.28)**^†^** |
| M99 | Biomechanical lesions, not elsewhere classified | 17 | 10.6 | 140 | 8.7 | 1.21 (0.75, 1.96) |
| T78 | Adverse effects, not elsewhere classified | 17 | 10.6 | 109 | 6.8 | 1.56 (0.96, 2.53) |
| D22 | Melanocytic nevi | 17 | 10.6 | 143 | 8.9 | 1.19 (0.74, 1.91) |
| J45 | Asthma | 16 | 9.9 | 115 | 7.1 | 1.39 (0.85, 2.29) |
| E03 | Other hypothyroidism | 16 | 9.9 | 126 | 7.8 | 1.27 (0.77, 2.08) |
| J03 | Acute tonsillitis | 16 | 9.9 | 94 | 5.8 | 1.70 (1.03, 2.82)**^†^** |
| Q66 | Congenital deformities of feet | 15 | 9.3 | 99 | 6.1 | 1.52 (0.90, 2.54) |
| T14 | Injury of unspecified body region | 15 | 9.3 | 91 | 5.7 | 1.65 (0.98, 2.78) |
| Z00 | Encounter for general exam without complaint, suspected or reported dx | 15 | 9.3 | 105 | 6.5 | 1.43 (0.85, 2.39) |
| K29 | Gastritis and duodenitis | 14 | 8.7 | 125 | 7.8 | 1.12 (0.66, 1.90) |
| E78 | Disorders of lipoprotein metabolism and other lipidemias | 14 | 8.7 | 76 | 4.7 | 1.84 (1.07, 3.18)**^†^** |
| I10 | Essential primary hypertension | 14 | 8.7 | 103 | 6.4 | 1.36 (0.80, 2.32) |
| M25 | Other joint disorder, not elsewhere classified | 13 | 8.1 | 89 | 5.5 | 1.46 (0.84, 2.55) |
| Z27 | Need for immunization against combinations of infectious diseases | 13 | 8.1 | 73 | 4.5 | 1.78 (1.01, 3.14)**^†^** |
| M53 | Other and unspecified dorsopathies, not elsewhere classified | 13 | 8.1 | 113 | 7.0 | 1.15 (0.66, 2.00) |
| J20 | Acute bronchitis | 13 | 8.1 | 122 | 7.6 | 1.07 (0.62, 1.84) |
| G43 | Migraine | 13 | 8.1 | 121 | 7.5 | 1.07 (0.62, 1.86) |
| B34 | Viral infection of unspecified site | 13 | 8.1 | 87 | 5.4 | 1.49 (0.85, 2.62) |
| K52 | Other and unspecified noninfective gastroenteritis and colitis | 12 | 7.5 | 98 | 6.1 | 1.22 (0.69, 2.18) |
| M51 | Thoracic, thoracolumbar, and lumbosacral intervertebral disc disorders | 12 | 7.5 | 55 | 3.4 | 2.18 (1.19, 3.99)**^†^** |
| L70 | Acne | 12 | 7.5 | 109 | 6.8 | 1.10 (0.62, 1.95) |
| J02 | Acute pharyngitis | 12 | 7.5 | 114 | 7.1 | 1.05 (0.59, 1.87) |
| Z71 | Persons encounter health services for other counseling and medical advice, not elsewhere classified | 11 | 6.8 | 47 | 2.9 | 2.34 (1.24, 4.42)**^†^** |
| F33 | Major depressive disorder, recurrent | 11 | 6.8 | 53 | 3.3 | 2.08 (1.11, 3.89)**^†^** |
| M41 | Scoliosis | 11 | 6.8 | 54 | 3.4 | 2.04 (1.09, 3.82)**^†^** |
| N39 | Other disorders of urinary system | 11 | 6.8 | 85 | 5.3 | 1.29 (0.71, 2.37) |
| I95 | Hypotension | 10 | 6.2 | 36 | 2.2 | 2.78 (1.40, 5.49)**^†^** |
| Z26 | Necessity of immunization against other single infectious diseases | 10 | 6.2 | 80 | 5.0 | 1.25 (0.66, 2.36) |
| R42 | Dizziness and giddiness | 10 | 6.2 | 49 | 3.0 | 2.04 (1.05, 3.95)**^†^** |
| R11 | Nausea and vomiting | 10 | 6.2 | 63 | 3.9 | 1.59 (0.83, 3.03) |
| L30 | Other and unspecified dermatitis | 10 | 6.2 | 82 | 5.1 | 1.22 (0.65, 2.30) |

^†^statistically significant

**Table S6.** ATC code and subcode of the most commonly prescribed agents in the early-diagnosed PKU cohort

| ATC code or subcode | Category or subcategory | Early-diagnosed  PKU cohort (n=161)  % | Control cohort (n=1,610)  % | PR (95% CI) |
| --- | --- | --- | --- | --- |
| **J** | **Antiinfectives for systemic use** | **34.8** | **34.7** | **1.00 (0.80, 1.25)** |
| J01 | Antibacterials for systemic use | 32.9 | 33.9 | 0.97 (0.77, 1.22) |
| J05 | Antivirals for systemic use | <5 | 0.6 | N/A |
| J07 | Vaccines | <5 | 0.3 | N/A |
| J06 | Immune sera and immunoglobulins | <5 | <5 | N/A |
| J02 | Antimycotics for systemic use | <5 | 0.5 | N/A |
| **N** | **Nervous system** | **26.7** | **17.8** | **1.50 (1.14, 1.98)^†^** |
| N06 | Psychoanaleptics | 14.3 | 6.1 | 2.35 (1.54, 3.59)**^†^** |
| N02 | Analgesics | 12.4 | 11.3 | 1.10 (0.71, 1.69) |
| N05 | Psycholeptics | 8.7 | 2.2 | 3.89 (2.14, 7.06)**^†^** |
| N03 | Antiepileptics | 3.7 | 0.7 | 5.00 (1.90, 13.14)**^†^** |
| N07 | Other nervous system drugs | <5 | 0.6 | N/A |
| N01 | Anesthetics | <5 | <5 | N/A |
| **M** | **Musculo-skeletal system** | **25.5** | **22.5** | **1.13 (0.86, 1.50)** |
| M01 | Antiinflammatory and antirheumatic products | 24.8 | 21.6 | 1.15 (0.86, 1.53) |
| M03 | Muscle relaxants | <5 | 2.1 | N/A |
| M04 | Antigout preparations | <5 | <5 | N/A |
| M05 | Drugs for treatment of bone diseases | <5 | 0.0 | N/A |
| **A** | **Alimentary tract and metabolism** | **24.8** | **14.0** | **1.78 (1.32, 2.39)^†^** |
| A02 | Drugs for acid related disorders | 16.1 | 9.3 | 1.73 (1.18, 2.54)**^†^** |
| A11 | Vitamins | 5.6 | 0.6 | 9.00 (3.71, 21.83)**^†^** |
| A12 | Minerals | 4.3 | <5 | N/A |
| A03 | Drugs for functional gastrointestinal disorders | <5 | 3.0 | N/A |
| A06 | Drugs for constipation | <5 | 0.4 | N/A |
| A01 | Stomatological preparations | <5 | 0.9 | N/A |
| A16 | Other alimentary tract and metabolism products | <5 | 0.0 | N/A |
| A04 | Antiemetics and antinauseants | <5 | <5 | N/A |
| **H** | **Systemic hormonal preparations, excl. sexual hormones and insulins** | **16.8** | **12.7** | **1.32 (0.91, 1.90)** |
| H03 | Thyroid therapy | 12.4 | 9.4 | 1.32 (0.86, 2.05) |
| H02 | Corticosteroids for systemic use | 6.8 | 3.4 | 2.00 (1.07, 3.74)**^†^** |
| **R** | **Respiratory system** | **15.5** | **15.1** | **1.03 (0.70, 1.50)** |
| R03 | Drugs for obstructive airway diseases | 6.2 | 7.2 | 0.86 (0.46, 1.61) |
| R01 | Rhinologics | 6.2 | 5.0 | 1.23 (0.65, 2.33) |
| R05 | Cough and cold preparations | 3.7 | 2.9 | 1.30 (0.57, 3.01) |
| R06 | Antihistamines for systemic use | 3.1 | 2.5 | 1.22 (0.49, 3.04) |
| **D** | **Dermatologicals** | **13.0** | **11.3** | **1.15 (0.76, 1.76)** |
| D07 | Corticosteroide, dermatological preparations | 7.5 | 6.1 | 1.22 (0.69, 2.18) |
| D01 | Antimycotics for dermatological use | <5 | 2.0 | N/A |
| D06 | Antibiotics and chemotherapeutics for dermatological use | <5 | 2.0 | N/A |
| D11 | Other dermatological preparations | <5 | 0.4 | N/A |
| D08 | Antiseptics and disinfectants | <5 | <5 | N/A |
| D10 | Antiacne preparations | <5 | 2.0 | N/A |
| D02 | Emollients and skin protectives | <5 | 0.0 | N/A |
| **C** | **Cardiovascular system** | **12.4** | **6.3** | **1.98 (1.26, 3.11)^†^** |
| C09 | Agents acting on the renin-angiotensin system | 6.2 | 3.4 | 1.85 (0.96, 3.57) |
| C07 | Beta blocking agents | 5.6 | 2.9 | 1.96 (0.98, 3.92) |
| C03 | Diuretics | 3.1 | 0.3 | 10.00 (2.93, 34.18)**^†^** |
| C08 | Calcium channel blockers | <5 | 0.6 | N/A |
| C10 | Lipid modifying agents | <5 | 0.4 | N/A |
| C02 | Antihypertensives | <5 | <5 | N/A |
| C05 | Vasoprotectives | <5 | 0.3 | N/A |
| C01 | Cardiac therapy | <5 | <5 | N/A |
| **G** | **Genitourinary system and sexual hormones** | **11.8** | **7.7** | **1.53 (0.97, 2.41)** |
| G03 | Sexual hormones and modulators of the genital system | 8.1 | 5.3 | 1.53 (0.87, 2.68) |
| G01 | Gynecological antiinfectives and antiseptics | 3.1 | 2.4 | 1.28 (0.51, 3.21) |
| G04 | Urologicals | <5 | 0.5 | N/A |
| G02 | Other gynecologicals | <5 | <5 | N/A |
| **B** | **Blood and blood forming organs** | **7.5** | **3.5** | **2.11 (1.15, 3.84)^†^** |
| B01 | Antithrombotic agents | 3.7 | 1.7 | 2.14 (0.90, 5.10) |
| B03 | Antianemic preperations | 3.1 | 1.8 | 1.72 (0.68, 4.39) |
| B05 | Blood substitutes and perfusion solutions | <5 | <5 | N/A |
| **S** | **Sensory organs** | **6.2** | **7.4** | **0.84 (0.45, 1.57)** |
| S01 | Ophthalmologicals | 5.0 | 6.1 | 0.82 (0.40, 1.65) |
| S02 | Otologicals | <5 | <5 | N/A |
| **L** | **Antineoplastic and immunomodulating agents** | **4.3** | **1.6** | **2.80 (1.23, 6.37)^†^** |
| L04 | Immunosuppressants | <5 | 0.7 | N/A |
| L01 | Antineoplastic agents | <5 | 0.6 | N/A |
| L03 | Immunostimulants | <5 | <5 | N/A |
| **V** | **Various** | **3.1** | **1.7** | **1.79 (0.70, 4.56)** |
| V07 | All other non-therapeutic products | <5 | 0.0 | N/A |
| V01 | Allergens | <5 | 0.7 | N/A |
| V04 | Diagnostic agents | <5 | 0.9 | N/A |
| **P** | **Antiparasitic products, insecticides and repellents** | **<5** | **1.4** | **N/A** |
| P01 | Antiprotozoals | <5 | 0.8 | N/A |
| P03 | Ectoparasiticides, incl. scabicides, insecticides and repellents | <5 | <5 | N/A |

^†^statistically significant

**Table S7.** Top 50 most prevalent comorbidities of the late-diagnosed PKU cohort and the corresponding prevalence in the control cohort in Germany in 2015

| ICD-10-GM code | Description | Late-diagnosed  PKU cohort (n=216)  % | Control cohort (n=2,160)  % | PR (95% CI) |
| --- | --- | --- | --- | --- |
| I10 | Essential primary hypertension | 61.1 | 58.4 | 1.05 (0.94, 1.17) |
| E78 | Disorders of lipoprotein metabolism and other lipidemias | 52.3 | 40.4 | 1.30 (1.13, 1.49)**^†^** |
| M54 | Dorsalgia | 42.1 | 36.1 | 1.17 (0.99, 1.38) |
| H52 | Disorders of refraction and accommodation | 38.0 | 33.1 | 1.15 (0.96, 1.37) |
| Z12 | Encounter for screening for malignant neoplasms | 28.2 | 30.0 | 0.94 (0.75, 1.18) |
| I25 | Chronic ischemic heart disease | 25.9 | 15.6 | 1.67 (1.30, 2.13)**^†^** |
| E11 | Type 2 diabetes mellitus | 25.5 | 19.6 | 1.30 (1.02, 1.65)**^†^** |
| Z25 | Need for immunization against other single viral diseases | 22.2 | 20.4 | 1.09 (0.84, 1.42) |
| M17 | Osteoarthritis of knee | 21.3 | 17.2 | 1.24 (0.94, 1.63) |
| Z00 | Encounter for general exam without complaint. suspected or reported dx | 20.8 | 20.2 | 1.03 (0.79, 1.36) |
| N95 | Menopausal and other perimenopausal disorders | 20.4 | 14.5 | 1.40 (1.06, 1.86)**^†^** |
| E04 | Other nontoxic goiter | 19.9 | 14.7 | 1.35 (1.02, 1.80)**^†^** |
| M47 | Spondylosis | 19.4 | 18.8 | 1.03 (0.78, 1.37) |
| E66 | Overweight and obesity | 19.0 | 14.2 | 1.34 (0.99, 1.79) |
| F32 | Major depressive disorder, single episode | 19.0 | 15.8 | 1.20 (0.90, 1.61) |
| M53 | Other and unspecified dorsopathies, not elsewhere classified | 19.0 | 12.6 | 1.51 (1.12, 2.03)**^†^** |
| Z01 | Encounter for other specified exam without complaint, suspected or reported dx | 18.5 | 12.9 | 1.43 (1.06, 1.94)**^†^** |
| E14 | Unspecified diabetes mellitus | 18.5 | 10.7 | 1.73 (1.28, 2.35)**^†^** |
| E79 | Disorders of purine and pyrimidine metabolism | 17.6 | 11.1 | 1.59 (1.16, 2.17)**^†^** |
| N39 | Other disorders of urinary system | 17.6 | 12.5 | 1.40 (1.03, 1.91)**^†^** |
| Z92 | Personal history of medical treatment | 17.1 | 13.0 | 1.32 (0.97, 1.81) |
| F45 | Somatoform disorders | 17.1 | 14.3 | 1.20 (0.88, 1.64) |
| J44 | Other chronic obstructive pulmonary disease | 16.2 | 8.2 | 1.97 (1.41, 2.75)**^†^** |
| J06 | Acute upper respiratory infections of multiple and unspecified sites | 15.7 | 12.2 | 1.29 (0.93, 1.79) |
| K29 | Gastritis and duodenitis | 15.7 | 11.1 | 1.42 (1.02, 1.98)**^†^** |
| R42 | Dizziness and giddiness | 14.8 | 8.3 | 1.79 (1.26, 2.53)**^†^** |
| I83 | Varicose veins of lower extremities | 14.8 | 13.0 | 1.14 (0.82, 1.60) |
| Z96 | Presence of other functional implants | 14.4 | 14.7 | 0.97 (0.69, 1.37) |
| N89 | Other noninflammatory disorders of vagina | 14.4 | 10.6 | 1.35 (0.96, 1.92) |
| M19 | Other and unspecified osteoarthritis | 14.4 | 10.1 | 1.42 (1.00, 2.01) |
| H35 | Other retinal disorders | 13.9 | 11.3 | 1.23 (0.86, 1.75) |
| L30 | Other and unspecified dermatitis | 13.9 | 9.0 | 1.54 (1.08, 2.20)**^†^** |
| I70 | Atherosclerosis | 13.9 | 7.6 | 1.82 (1.26, 2.61)**^†^** |
| J45 | Asthma | 13.4 | 6.9 | 1.93 (1.33, 2.81)**^†^** |
| I50 | Heart failure | 13.4 | 8.3 | 1.61 (1.12, 2.32)**^†^** |
| M21 | Other acquired deformities of limbs | 13.0 | 5.0 | 2.59 (1.75, 3.83)**^†^** |
| E03 | Other hypothyroidism | 13.0 | 10.7 | 1.21 (0.84, 1.75) |
| N18 | Chronic kidney disease | 13.0 | 7.9 | 1.64 (1.13, 2.38)**^†^** |
| N40 | Enlarged prostate | 13.0 | 12.3 | 1.06 (0.73, 1.52) |
| K76 | Other diseases of liver | 12.5 | 10.6 | 1.18 (0.82, 1.72) |
| M16 | Osteoarthritis of hip | 12.5 | 8.9 | 1.41 (0.96, 2.05) |
| H25 | Age-related cataract | 12.5 | 11.2 | 1.12 (0.77, 1.62) |
| D22 | Melanocytic nevi | 12.5 | 9.4 | 1.34 (0.92, 1.95) |
| I49 | Other cardiac arrhythmias | 12.0 | 10.0 | 1.20 (0.82, 1.76) |
| Z95 | Presence of cardiac and vascular implants and grafts | 12.0 | 8.1 | 1.48 (1.00, 2.18) |
| M51 | Thoracic. thoracolumbar and lumbosacral intervertebral disc disorders | 11.6 | 12.8 | 0.91 (0.62, 1.33) |
| R10 | Abdominal and pelvic pain | 11.6 | 10.1 | 1.14 (0.77, 1.69) |
| M42 | Spinal osteochondrosis | 11.6 | 8.5 | 1.36 (0.92, 2.01) |
| H61 | Other disorders of external ear | 11.6 | 10.5 | 1.11 (0.75, 1.63) |

^†^statistically significant

**Table S8.** ATC code and subcode of the most commonly prescribed agents in the late-diagnosed PKU cohort

| ATC code or subcode | Category or subcategory | Late-diagnosed PKU cohort (n=216)  % | Control cohort (n=2,160)  % | PR (95% CI) |
| --- | --- | --- | --- | --- |
| **C** | **Cardiovascular system** | **67.1** | **60.6** | **1.11 (1.00, 1.22)** |
| C09 | Agents acting on the renin-angiotensin system | 47.7 | 44.7 | 1.07 (0.92, 1.24) |
| C07 | Beta blocking agents | 39.4 | 32.6 | 1.21 (1.01, 1.44)**^†^** |
| C10 | Lipid modifying agents | 32.4 | 22.5 | 1.44 (1.17, 1.77)**^†^** |
| C03 | Diuretics | 25.5 | 17.9 | 1.42 (1.11, 1.82)**^†^** |
| C08 | Calcium channel blockers | 19.9 | 15.1 | 1.32 (0.99, 1.76) |
| C01 | Cardiac therapy | 10.2 | 5.4 | 1.90 (1.23, 2.93)**^†^** |
| C02 | Antihypertensives | 2.3 | 3.1 | 0.74 (0.30, 1.80) |
| C05 | Vasoprotectives | <5 | 1.0 | N/A |
| C04 | Peripheral vasodilators | <5 | <5 | N/A |
| **A** | **Alimentary tract and metabolism** | **52.3** | **41.3** | **1.27 (1.10, 1.45)^†^** |
| A02 | Drugs for acid related disorders | 39.4 | 28.3 | 1.39 (1.16, 1.66)**^†^** |
| A10 | Antidiabetics | 15.7 | 13.4 | 1.17 (0.85, 1.63) |
| A11 | Vitamines | 3.7 | 3.0 | 1.23 (0.60, 2.53) |
| A03 | Drugs for functional gastrointestinal disorders | 3.7 | 4.2 | 0.89 (0.44, 1.81) |
| A12 | Minerals | 3.2 | 2.2 | 1.49 (0.68, 3.25) |
| A07 | Antidiarrheals, intestinal antiinflammatory/ antiinfective agents | 2.8 | 1.5 | 1.82 (0.77, 4.29) |
| A01 | Stomatological | 2.3 | 1.3 | 1.72 (0.67, 4.41) |
| A06 | Drugs for constipation | <5 | 2.9 | N/A |
| A04 | Antiemetics and antinauseants | <5 | 0.8 | N/A |
| A16 | Other alimentary tract and metabolism products | <5 | 0.0 | N/A |
| A09 | Digestives, incl. enzymes | <5 | 0.3 | N/A |
| **N** | **Nervous system** | **50.5** | **36.3** | **1.39 (1.20, 1.60)^†^** |
| N02 | Analgesics | 33.3 | 24.8 | 1.35 (1.10, 1.65)**^†^** |
| N06 | Psychoanaleptics | 19.4 | 12.0 | 1.62 (1.20, 2.17)**^†^** |
| N05 | Psycholeptics | 8.8 | 7.8 | 1.13 (0.72, 1.78) |
| N03 | Antiepileptics | 6.5 | 4.5 | 1.43 (0.83, 2.46) |
| N04 | Anti-parkinson drugs | 3.7 | 1.9 | 1.90 (0.91, 4.00) |
| N07 | Other nervous system drugs | 3.2 | 1.8 | 1.84 (0.83, 4.07) |
| N01 | Anesthetics | <5 | <5 | N/A |
| **M** | **Musculo-skeletal system** | **42.6** | **39.2** | **1.09 (0.92, 1.28)** |
| M01 | Antiinflammatory and antirheumatic products | 32.9 | 31.9 | 1.03 (0.84, 1.26) |
| M04 | Antigout preparations | 13.0 | 8.3 | 1.56 (1.08, 2.27)**^†^** |
| M05 | Drugs for treatment of bone diseases | 3.7 | 2.6 | 1.43 (0.69, 2.96) |
| M03 | Muscle relaxants | <5 | 1.7 | N/A |
| **J** | **Antiinfectives for systemic use** | **38.0** | **33.5** | **1.13 (0.95, 1.36)** |
| J01 | Antibacterials for systemic use | 36.1 | 32.0 | 1.13 (0.93, 1.36) |
| J07 | Vaccines | <5 | 0.3 | N/A |
| J05 | Antivirals for systemic use | <5 | 1.9 | N/A |
| J06 | Immune sera and immunoglobulins | <5 | <5 | N/A |
| J02 | Antimycotics for systemic use | <5 | 0.5 | N/A |
| **H** | **Systemic hormonal preparations, excl. sexual hormones and insulins** | **30.6** | **26.5** | **1.15 (0.93, 1.43)** |
| H03 | Thyroid therapy | 19.9 | 19.7 | 1.01 (0.76, 1.34) |
| H02 | Corticosteroids for systemic use | 13.0 | 9.4 | 1.37 (0.95, 1.99) |
| H04 | Pancreatic hormones | <5 | <5 | N/A |
| H05 | Calcium homeostasis | <5 | <5 | N/A |
| H01 | Pituitary and hypothalamic hormones and analogues | <5 | <5 | N/A |
| **D** | **Dermatologicals** | **28.7** | **18.8** | **1.53 (1.22, 1.92)^†^** |
| D07 | Corticosteroide, dermatological preparations | 16.2 | 11.2 | 1.45 (1.05, 2.01)**^†^** |
| D01 | Antimycotics for dermatological use | 7.9 | 4.7 | 1.68 (1.03, 2.76)**^†^** |
| D06 | Antibiotics and chemotherapeutics for dermatological use | 4.6 | 2.5 | 1.85 (0.96, 3.58) |
| D11 | Other dermatological preparations | <5 | 1.6 | N/A |
| D03 | Preparations for treatment of wounds and ulcers | <5 | <5 | N/A |
| D09 | Medical bandages | <5 | 1.1 | N/A |
| D05 | Antipsoriatics | <5 | 0.8 | N/A |
| D08 | Antiseptics and disinfectants | <5 | 0.4 | N/A |
| D10 | Antiacne preparations | <5 | 0.5 | N/A |
| **R** | **Respiratory system** | **25.9** | **17.6** | **1.47 (1.15, 1.87)^†^** |
| R03 | Drugs for obstructive airway diseases | 18.1 | 11.8 | 1.53 (1.13, 2.08)**^†^** |
| R05 | Cough and cold preparations | 6.0 | 3.6 | 1.69 (0.95, 2.99) |
| R01 | Rhinologics | 5.6 | 3.4 | 1.62 (0.90, 2.94) |
| R06 | Antihistamines for systemic use | 3.2 | 2.2 | 1.49 (0.68, 3.25) |
| **B** | **Blood and blood forming organs** | **22.7** | **22.6** | **1.00 (0.77, 1.30)** |
| B01 | Antithrombotic agents | 20.4 | 19.7 | 1.03 (0.78, 1.36) |
| B03 | Antianemic preperations | 3.7 | 3.8 | 0.98 (0.48, 1.99) |
| B05 | Blood substitutes and perfusion solutions | 2.3 | 1.6 | 1.47 (0.58, 3.72) |
| **G** | **Genitourinary system and sexual hormones** | **21.3** | **14.4** | **1.47 (1.12, 1.94)^†^** |
| G04 | Urologicals | 12.0 | 8.6 | 1.40 (0.95, 2.06) |
| G03 | Sexual hormones and modulators of the genital system | 7.9 | 6.0 | 1.31 (0.80, 2.13) |
| G01 | Gynecological antiinfectives and antiseptics | 3.2 | 0.6 | 5.38 (2.17, 13.35)**^†^** |
| G02 | Other gynecologicals | <5 | <5 | <5 |
| **S** | **Sensory organs** | **10.6** | **14.4** | **0.74 (0.50, 1.11)** |
| S01 | Ophthalmologicals | 8.8 | 13.3 | 0.66 (0.42, 1.03) |
| S02 | Otologicals | <5 | 1.1 | N/A |
| **V** | **Various** | **9.3** | **6.7** | **1.39 (0.89, 2.17)** |
| V04 | Diagnostic agents | 8.3 | 5.7 | 1.46 (0.91, 2.35) |
| V03 | All other therapeutic products | <5 | 0.6 | N/A |
| V08 | Contrast media | <5 | <5 | N/A |
| V01 | Allergens | <5 | <5 | N/A |
| **L** | **Antineoplastic and immunomodulating agents** | **4.6** | **4.9** | **0.94 (0.50, 1.78)** |
| L01 | Antineoplastic agents | 3.2 | 2.2 | 1.49 (0.68, 3.25) |
| L02 | Endocrine therapy | <5 | 2.1 | N/A |
| L04 | Immunosuppressants | <5 | 1.1 | N/A |
| L03 | Immunostimulants | <5 | 0.3 | N/A |
| **P** | **Antiparasitic products, insecticides and repellents** | **<5** | **1.7** | **N/A** |
| P01 | Antiprotozoals | <5 | 1.6 | N/A |

^†^statistically significant
